# ﻿Identification and reproductive isolation of *Euborellia* species (Insecta, Dermaptera, Anisolabididae) from East and Southeast Asia

**DOI:** 10.3897/zookeys.1146.98248

**Published:** 2023-02-07

**Authors:** Yoshitaka Kamimura, Chow-Yang Lee, Junsuke Yamasako, Masaru Nishikawa

**Affiliations:** 1 Department of Biology, Keio University, Yokohama 223-8521, Japan Universiti Sains Malaysia Penang Malaysia; 2 Urban Entomology Laboratory, Vector Control Research Unit, School of Biological Sciences, Universiti Sains Malaysia, Minden 11800, Penang, Malaysia Keio University Yokohama Japan; 3 Present address: Department of Entomology, University of California, Riverside, CA 92521, USA University of California Riverside United States of America; 4 Institute for Plant Protection, NARO, Kannondai 3-1-3, Tsukuba, Ibaraki 305-8604, Japan Institute for Plant Protection, NARO Tsukuba Japan; 5 Entomological Laboratory, Faculty of Agriculture, Ehime University, Tarumi 3-5-7, Matsuyama 790-8566, Japan Ehime University Matsuyama Japan

**Keywords:** DNA barcoding, *Euborelliapallipes*, genital morphology, post-copulatory reproductive isolation, reinstatement

## Abstract

*Euborellia* (Anisolabididae: Anisolabidinae) is one of the most speciose genera of earwigs (Dermaptera), and its species-level classification is difficult. To settle the classification of brachypterous species with abbreviated tegmina recorded from East and Southeast Asia, we examined the morphology and reproductive isolation of three tentative *Euborellia* species, and analyzed the DNA barcoding region of the mitochondrial cytochrome oxidase subunit I (COI) gene. The observed complete reproductive isolation among the three *Euborellia* taxa and considerable differentiation in the COI sequences clearly show that each should be treated as a separate species. Based on morphology, distribution and the DNA sequence, we identify *Euborellia* sp. 1 of Malaysia as *E.annulata* (Fabricius), a circumtropical cosmopolitan with no records of a fully winged form. Samples from Ioto Island (= Iwo-jima Island: Ogasawara Islands, southern Japan) were also identified as this species. *Euborellia* sp. 3, from the main islands of Japan, was generally larger and lacked a Y-shaped pigmented area on the penis lobe, which is characteristic of *Euborellia* sp. 1. We propose reinstating *E.pallipes* (Shiraki) as the oldest name for this taxon. *Euborellia* sp. 2, even the brachypterous form, can be distinguished from these two species by its paler coloration (particularly the femora), ecarinate post-abdomen, and the shape of the male genitalia (parameres). We tentatively identify this species as *E.philippinensis* Srivastava based on the morphology of the brachypterous form, although the macropterous form cannot be distinguished from *E.femoralis* (Dohrn).

## ﻿Introduction

*Euborellia* Burr, 1910 (Anisolabididae: Anisolabidinae) is one of the most speciose genera of earwigs. It includes approximately 50 species (Popham and Brindle 1966; Sakai 1982, 1987; Steinmann 1989a, b; Srivastava 1999; Hopkins et al. 2018), in a small order of polyneopteran insects (Insecta: Dermaptera) with more than 2000 described species (Zhang 2013; Hopkins et al. 2018). Like related genera of Anisolabidinae, many *Euborellia* are apterous or brachypterous and are usually dark in color. Due to the scarcity of traits for species diagnoses, classifying this genus is very difficult.

This study examined the relationships among three tentative *Euborellia* species with flap-like vestigial tegmina (= forewings) found in East and Southeast Asia (named *Euborellia* sp. 1, *Euborellia* sp. 2 and *Euborellia* sp. 3 in the rest of the article). *Euborellia* sp. 1 was recorded from Malaysia, and was tentatively identified as *Euborelliaannulata* (Fabricius, 1793) in Kamimura et al. (2016). Although the type locality of *E.annulata* is the West Indies, many authors consider this species a senior synonym of *Euborelliastali* (Dohrn, 1864), the type locality of which is Java, which makes it a circumtropical cosmopolitan (Brindle 1981; Sakai 1987; Srivastava 2003). Except for doubtful treatments of this species as a synonym of other *Euborellia* species with fully developed tegmina and wings (see the Results and discussion), both male and female adults of this species are brachypterous. This means that they only have vestigial tegmina as small oval flaps and entirely lack hindwings (Dohrn 1864; Brindle 1981; Sakai 1987; Srivastava 2003; but see Kamimura et al. 2016 for a single aberrant laboratory-raised male with fully developed tegmina but no hindwings). *Euborellia* sp. 2 is known only at the west coast of Penang Island in Malaysia (Kamimura et al. 2016). Although in that study all wild-caught samples were brachypterous, macropterous individuals with fully developed tegmina and wings were found in laboratory-reared populations. Based on the morphology of the brachypterous morph, the previous study tentatively identified the species as *Euborelliaphilippinensis* Srivastava, 1979, considered endemic to the Philippines (Srivastava 1979, 1999; Sakai 1987; Steinmann 1989a, b). A third possibly distinct species of brachypterous *Euborellia*, tentatively named *Euborellia* sp. 3 here, occurs in the temperate zone of Japan. These three species are inhabiting open lands, including agricultural fields, semi-urban grasslands, sandy seaside or streamside, and can be collected by hand-sorting (Kamimura et al. 2016; Nishikawa 2016).

An apterous species of *Euborellia* was recently discovered as a possible intruder in Europe (Kalaentzis et al. 2021). Based on both morphological and molecular evidence, this species was identified as the apterous form of the Oriental species *Euborelliafemoralis* (Dohrn, 1863), which is usually macropterous (Kalaentzis et al. 2021). To resolve the cryptic species diversity of Anisolabidinae in Australia, Stuart et al. (2019) also demonstrated the effectiveness of an approach incorporating both morphometric and molecular analyses. To settle the classification of *Euborellia* species in Asia, we thus examined reproductive isolation among the three tentative species (*Euborellia* sp. 1, 2, and 3), and their detailed external and genital morphologies. Based on sequences of a mitochondrial cytochrome oxidase subunit I (COI) gene region, which is widely used for DNA barcoding of Dermaptera (Matzke and Kočárek 2015; Stuart et al. 2019; Kalaentzis et al. 2021; Kočárek and Wahab 2021), the genetic divergence and phylogenetic relationships among these and other *Euborellia* species were also examined.

## ﻿Materials and methods

### ﻿Reproductive isolation

Two experiments examined pre- and post-copulatory reproductive isolation among the three tentative species. Virgin females were obtained by separating newly emerged adults every three days from laboratory cultures of nymphs (wild-caught or mainly the F_1_ generation). For *Euborellia* sp. 1 and *Euborellia* sp. 3, individuals derived from five localities of Malaysia (Batu Ferringi [5.47°N, 100.25°E], Bukit Bendera [5.42°N, 100.26°E], Bayan Indah beach [5.34°N, 100.31°E], and Bayan Lepas [5.33°N, 100.31°E] of Penang Island and Kuantan [3.80°N, 103.34°E], Pahang state: and some of their hybrid F_1_) and three localities from Japan (Tokushima city, Tokushima Prefecture [34.12°N, 134.58°E], Yokohama city, Kanagawa Prefecture [35.51°N, 139.57°E], and Komae city, Tokyo Prefecture [35.63°N, 139.57°E]) were used, respectively. All samples of *Euborellia* sp. 2 were derived from a single locality (Sungai Nipah, Penang Island, Malaysia [5.32°N, 100.20°E]), but pairing of a male and a female from the same full-sib family was avoided. For males, wild-caught adults were also used (see Suppl. material 1 for further details). All animals were kept at 26 ± 1 °C (12 h photoperiod) and provided with water and unlimited amounts of commercial cat food.

In the first experiment (Exp. 1), a virgin female (age: 5–68 days after imaginal eclosion: median = 9 days) was paired with a conspecific or heterospecific male in a plastic container (50 × 32 mm, 12 mm high) with plaster of Paris at the base for 21 h (*N* = 5 for each species combination). Then the females were sacrificed by placing them in a freezer (−20 °C) for later examination of their insemination status. The spermatheca was dissected from the females in insect Ringer’s solution (0.9 g NaCl, 0.02 g CaCl_2_, 0.02 g KCl, and 0.02 g NaHCO_3_ in 100 mL water) under a stereomicroscope (EZ vision, Saxon, Guangzhou, China), and then examined under a light microscope (BX53 or CX21, Olympus, Tokyo; 40–400×). In the second experiment (Exp. 2), a virgin female (age: 3–83 days after imaginal eclosion: median = 6 days) was paired with a conspecific or heterospecific male in a separate plastic vessel (60 mm diameter, 40 mm high) for 72 h (*N* = 5 for each species combination). Then the females were reared separately in the vessel for 30 days after removing the male. Oviposition and hatching of offspring were checked every two or three days. The spermatheca of the females that produced no hatchlings during the observation period was examined for the presence of sperm, as described above. Females with at least one hatchling or sperm in the spermatheca were scored as “inseminated”.

### ﻿External and genital morphology

The external morphologies of dried adult materials were examined under a stereomicroscope (S8-APO; Wetzlar, Germany or SZX16; Olympus, Tokyo, Japan) and photographed using an Olympus Pen e-pl1s digital camera (Olympus). “Microscope mode” and “Focus-stacking sub-mode” of a Tough-TG5 digital camera (Olympus) were also used to obtain composite images of the external traits. The male genitalia were extracted from freeze-preserved, dried, or fresh specimens anesthetized with carbon dioxide under a stereomicroscope. After mounting on a glass slide with insect Ringer’s solution, they were observed and photographed under a light microscope (BX53, 100–400×; Olympus) equipped with an Olympus DP80 CCD camera or a differential interference contrast (DIC) microscope (BX53, 100–400×; Olympus) fitted with an Olympus Pen e-pl1s digital camera. Based on photographs taken under the DIC microscope, selected parts of each image in focus were composed using Combine ZP Image Stacking Software (Hadley 2008).

The samples were wild-caught from Penang Island (Bayan Lepas [Penang-1], Batu Ferringi [Penang-2], and Bayan Indah beach [Penang-3]) for *Euborellia* sp. 1. For *Euborellia* sp. 2 and *Euborellia* sp. 3, samples of laboratory stock populations, derived from a female collected from Sungai Nipah, Penang Island, Malaysia (in 2012), and Takasago city, Hyogo Prefecture, Japan [34.75°N, 134.80°E] (in 2018), respectively, were examined. For *Euborellia* sp. 3, wild-caught and mainly F_1_-generation offspring were also examined for the following seven localities of Japan: Satsuma-sendai city, Kagoshima Prefecture [31.81°N, 130.31°E: Kagoshima-1], Shimokoshiki Island, Kagoshima Prefecture [31.66°N, 129.72°E: Kagoshima-2], Naruto city, Tokushima Prefecture [34.20°N, 134.60°E], Shizuoka city, Shizuoka Prefecture [35.01°N, 138.39°E: Shizuoka-1], Izunokuni city, Shizuoka Prefecture [35.06°N, 138.95°E: Shizuoka-2], Yokohama city, Kanagawa Prefecture [35.51°N, 139.57°E], and Iwaki city, Fukusima Prefecture [36.88°N, 140.79°E].

For *Euborellia* sp. 1 and 3, which were challenging to discriminate based on their external appearance, three traits were chosen for measurement based on the results of a pilot study: maximum head width (including eyes), maximum pronotum width, and hind tibia length. These traits can usually be measured on dried specimens preserved in museums and can be used for future studies on this group. These traits were measured for dried materials (Suppl. material 2) to the nearest 0.026 mm using a binocular microscope (SZ, Olympus) with an eyepiece. The mean values were used for subsequent analysis for samples in which both the right and left hind tibia lengths were measurable. Otherwise, the measurements of one side were used.

In addition to the samples collected by the authors, two female and one male adult *Euborellia* collected from Ioto Island (= Iwo-jima Island) in the Ogasawara Islands (= Bonin Islands) preserved in the collection of Kanagawa Prefectural Museum of Natural History (**KPMNH**), Japan were examined: 2♀♀, pond-side at the Northern Airfield site, Ioto Island, Ogasawara, Tokyo, 13–14.XII.2005, Haruki Karube leg.; 1♂, Ioto Island, Ogasawara, Tokyo, 31.XII.2004, Katsumi Sano leg. For comparison, an adult female sample of *E.annulata*, collected from French West Indies: Jarry, Basse-Terre Island, Guadeloupe Archipelago (16.23°N, 61.55°W: 20.XI.2020, Nicolas Moulin leg.) was also observed and measured. Holotype (female) of *Anisolabispallipes* Shiraki, 1905, in the collection of National Taiwan University (**NTU**), Taipei, Taiwan, was also examined onsite.

### ﻿DNA barcoding

Total genomic DNA was extracted from fresh, ethanol-preserved, or dried samples of *Euborellia* and other dermapterans (Suppl. material 3), using a DNeasy Blood & Tissue Kit (QIAGEN, Hilden, Germany) according to the manufacturer’s instructions. Depending on the size of the specimens, one to three legs on one side were used for DNA extraction. PCR amplification of a mitochondrial cytochrome oxidase subunit I (COI) region (660 base pairs), which is widely used for DNA barcoding of earwigs (Matzke and Kočárek 2015; Stuart et al. 2019; Kalaentzis et al. 2021; Kočárek and Wahab 2021) and other invertebrates (Folmer et al. 1994) was performed using a T100^TM^ thermal cycler (Bio-Rad Laboratories, Hercules, CA, USA) and primers LCO1490 and HCO2198 (Folmer et al. 1994). PCR reactions were conducted in a 20 μL volume containing 1 μL each primer (10 μM), 10 μL 2×PCR buffer, 4 μL dNTPs (2 mM each), 0.4 μL KOD FX Neo DNA polymerase (1.0 unit/μL; Toyobo, Osaka, Japan), and 1 μL genomic DNA. The PCR temperature profile consisted of 2 min at 94 °C, then 35 cycles of 15 sec at 94 °C, 15 sec at 51 °C, and 15 sec at 72 °C, followed by a 6 min final extension at 72 °C. Since the primer set did not work for *Euborelliaannulipes* (Lucas, 1847), another set of primers (SKCOI-7 and SKCOI-7) was used to obtain PCR products of this species, which largely overlapping with the LCO1490–HCO2198 region but lacking 44 bases of the 5’ end, according to the protocol of Su et al. (2004). Sequencing was done by Eurofins Genomics (Tokyo, Japan) (or FASMAC, Kanagawa, Japan for *E.annulipes*). The chromatograms were checked visually and edited manually where appropriate. After eliminating the primer sequences, the COI sequences have been deposited in DDBJ/ENA/GenBank.

Multiple sequence alignments were conducted with ClustalW (Thompson et al. 2003) implemented in MEGA11 (Tamura et al. 2021) using the default settings. The sequences of other *Euborellia* species and *Apachyusfeae* Bormans, 1894 (Apachyidae) available in GenBank were also included in the analysis (Accession numbers: MW703670.1, MW703671.1, MW703672.1, MW703673.1, MW291948.1, and KPO19208.2). The maximum likelihood (ML) analysis and calculation of intraspecific and interspecific *p*-distances were performed with MEGA11. For the ML analysis, the optimal nucleotide substitution model (the general time-reversible model [GTR]+G+I) was determined by MEGA11 using the Bayesian Information Criterion (BIC) and the default search algorithms: a discrete Gamma distribution (+G) with five rate categories and a certain fraction of sites evolutionarily invariable (+I). Because no non-dermapteran samples were added as outgroups, the resultant trees were rooted by a clade of the Infraorder Protodermaptera. Investigations of the sequence saturations were done plotting the estimated number of base substitutions (transitions and transversions) against the genetic distance (maximum composite likelihood model). The data were obtained for 741 comparisons of 38 sequences (660 bp) obtained in the present study by using MEGA11, and visualized by a personal script written in Python v.3.8.3.

## ﻿Results and discussion

### ﻿Reproductive isolation

**Figure 1.**
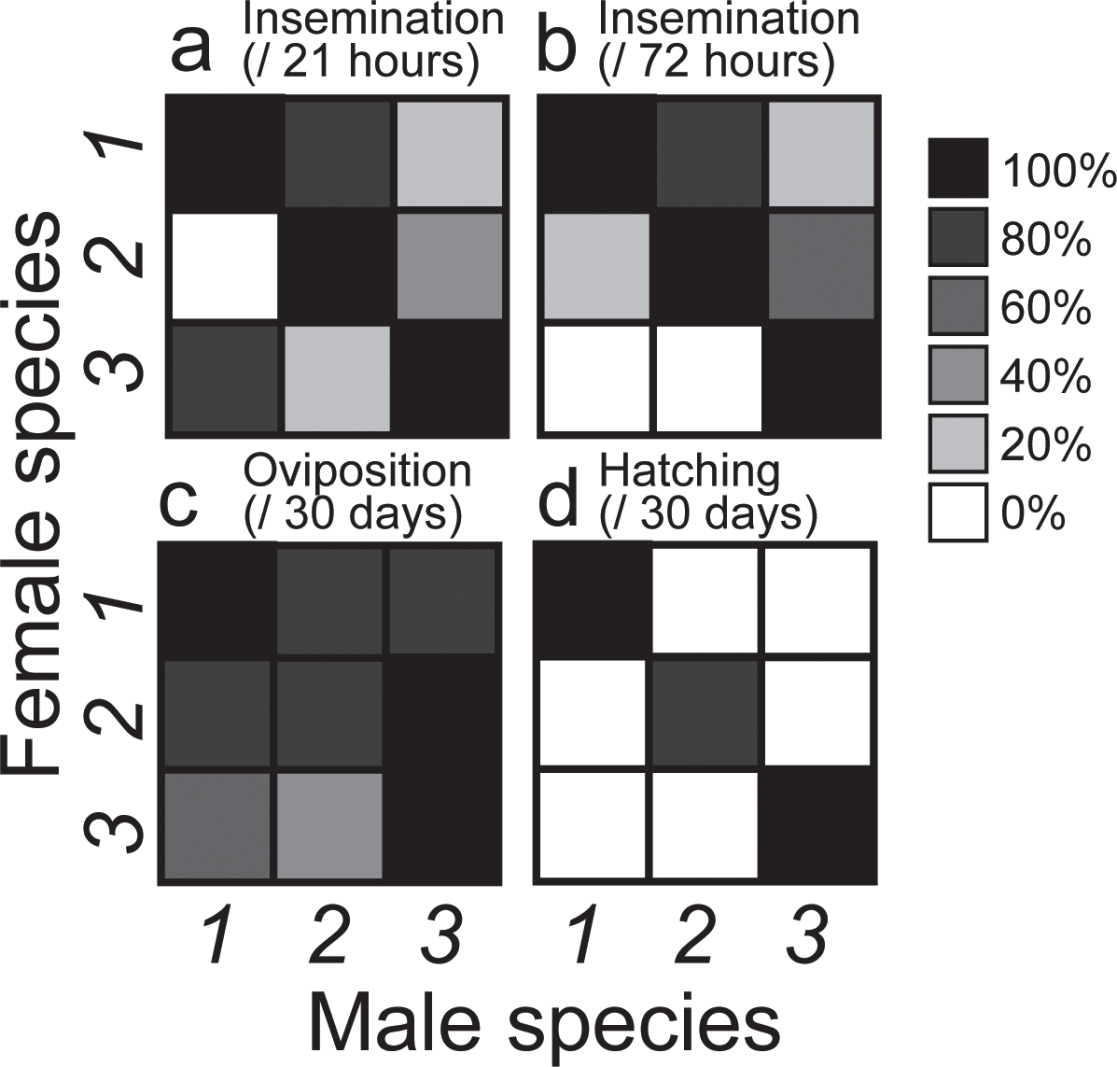
Insemination (**a, b**), oviposition (**c**), and hatching success (**d**) of conspecific and reciprocal heterospecific crosses among the three tentative species of *Euborellia*: *Euborellia* sp. 1 (*1*), *Euborellia* sp. 2 (*2*), and *Euborellia* sp. 3 (*3*). These species are identified as *E.annulata*, *E.philippinensis*, and *E.pallipes*, respectively.

The crossing experiments revealed that the three tentative *Euborellia* species are strongly isolated (Fig. 1). Interestingly, when the data for 21 h and 72 h pairings were combined, insemination was found to have occurred in all heterospecific pairing combinations (Fig. 1a, b). The insemination success between a female of *Euborellia* sp. 1 and a male of *Euborellia* sp. 2 was very high, 80% in both the 21 h and 72 h pairings (Fig. 1a, b). On average, 73.3% of females paired with a heterospecific male, and 93.3% paired with a conspecific male laid an egg batch (Fig. 1c). All egg batches of females paired with a conspecific male developed normally, resulting in the production of at least one hatchling (Fig. 1d). However, no development was observed in the eggs deposited after heterospecific pairings, with no hatchling success during the 30-day observation period (Fig. 1d).

### ﻿External and male genital morphology

**Figure 2.**
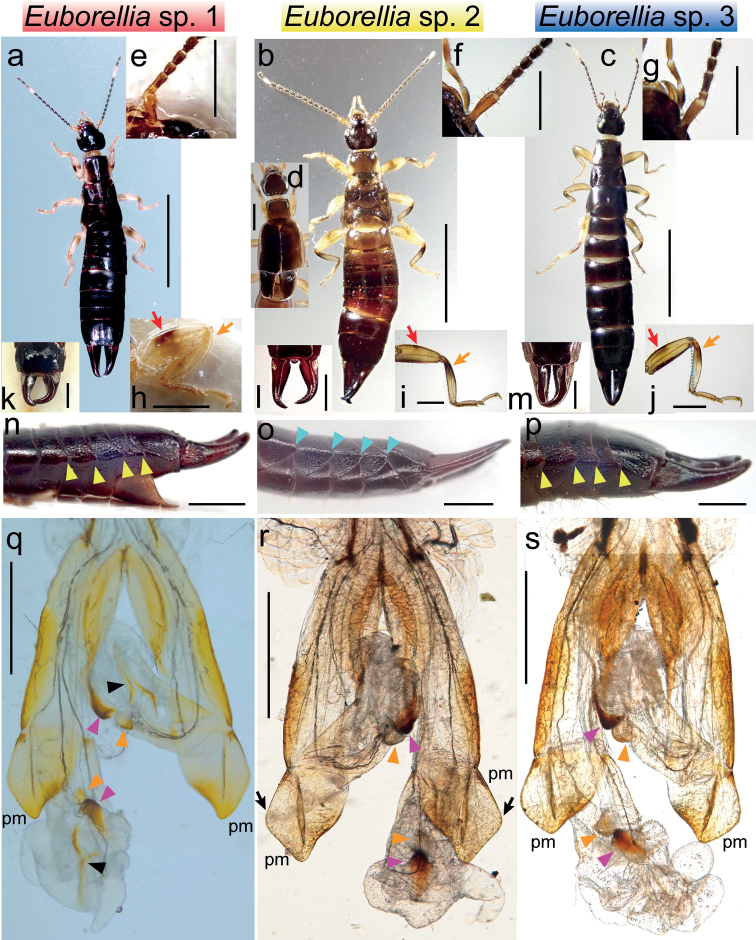
Female habitus (**a–c**), head and thorax of a fully winged-form male (**d**), base of female right antenna (**e–g**), female right hindleg (**h–j**), male forceps (**k–m**), left side of male post-abdomen (**n–p**), and distal part of male genitalia (**q–s**) of *Euborellia* sp. 1 (identified as *E.annulata*; **a, e, h, k, n, q**), *Euborellia* sp. 2 (identified as *E.philippinensis*; **b, d, f, i, l, o, r**), and *Euborellia* sp. 3 (identified as *E.pallipes*; **c, g, j, m, p, s**). Femoral and tibial black marking are indicated by the red and orange arrows, respectively (**h–j**). Carination and dorso-lateral angles of the abdominal tergites, the latter forms the lateral ridges, are indicated by the yellow and light blue arrowheads, respectively (**n–p**). On each penis lobe, a pair of denticulated pads (the orange and magenta arrowheads) and a Y-shaped area of pigmentation (only in *Euborellia* sp. 1: black arrowheads) are present. The external apical angle of the parameres (pm) is acute in *Euborellia* sp. 2 (the black arrow). Scale bars: 5 mm (**a–c**); 1 mm (**d–p**); 500 µm (**q–s**).

The external morphologies of the three tentative species, particularly those of *Euborellia* sp. 1 and 3, are similar and difficult to distinguish. Except for the fully winged morph of *Euborellia* sp. 2 (Fig. 2d), both male and female adults of these species have the tegmina abbreviated to small oval flaps (Fig. 2a–c). Kamimura et al. (2016) reported one aberrant male with fully developed tegmina but no hindwings for *Euborellia* sp. 1. The coloration of *Euborellia* sp. 2 is generally paler than the other two species, being dark brownish (Fig. 2a–d). In the legs of these species, a black marking develops in the mid part of the femur (indicated by red arrows in Fig. 2h–j) and in the basal half of the tibia (indicated by orange arrows in Fig. 2h–j). In *Euborellia* sp. 1, the former marking is much more conspicuous than the latter, forming an almost complete band (Fig. 2h). By contrast, the tibial marking is more prominent in *Euborellia* sp. 2 (Fig. 2i). These black markings of *Euborellia* sp. 3 develop at almost the same intensity. Still, the femoral band usually does not reach the ventral side (Fig. 2j).

Kalaentzis et al. (2021) reported that the relative lengths of the basal antennomeres are useful for diagnosing *Euborellia* species. However, we found no conspicuous difference in the antennal morphology of the three tentative species: the 1^st^ antennomere is as long as or slightly shorter than the length of the 2^nd^, 3^rd^, and 4^th^ combined (Fig. 2e–g). In males of *Euborellia* sp.1 and 3, the lateral sides of the abdominal segments 6^th^ (in some cases 7^th^) to 9^th^ are acute-angled posteriorly and carinated (Fig. 2n, p: yellow arrowheads). The corresponding abdominal tergites of *Euborellia* sp. 2 are bent at an almost right angle (Fig. 2o: light blue arrowheads), making the post-abdomen cross-sections rectangular.

The genital morphologies are also quite similar among the *Euborellia* species examined here. The male genitalia are elongated, almost the body length (*Euborellia* sp. 1 and 3) or the abdominal length (*Euborellia* sp. 2). On each penis lobe including a thin virga, two humps of denticulated pads are present (orange and magenta arrowheads in Fig. 2q–s). In addition, a conspicuous Y-shaped area of pigmentation is present only in *Euborellia* sp. 1 (Fig. 2q: black arrowhead). Although previous descriptions of *Euborellia* species lack such a detailed morphology of the penis lobes, judging from the high-resolution images in Kalaentzis et al. (2021), the penises of *E.femoralis* and *E.annulipes* lack the Y-shaped pigmentation. The shape of the parameres is also similar among the species, being weakly emarginated on the inner side. The outer margin is strongly angular in *Euborellia* sp. 2 compared to *Euborellia* sp. 1 and 3 (black arrows in Fig. 2r).

**Figure 3.**
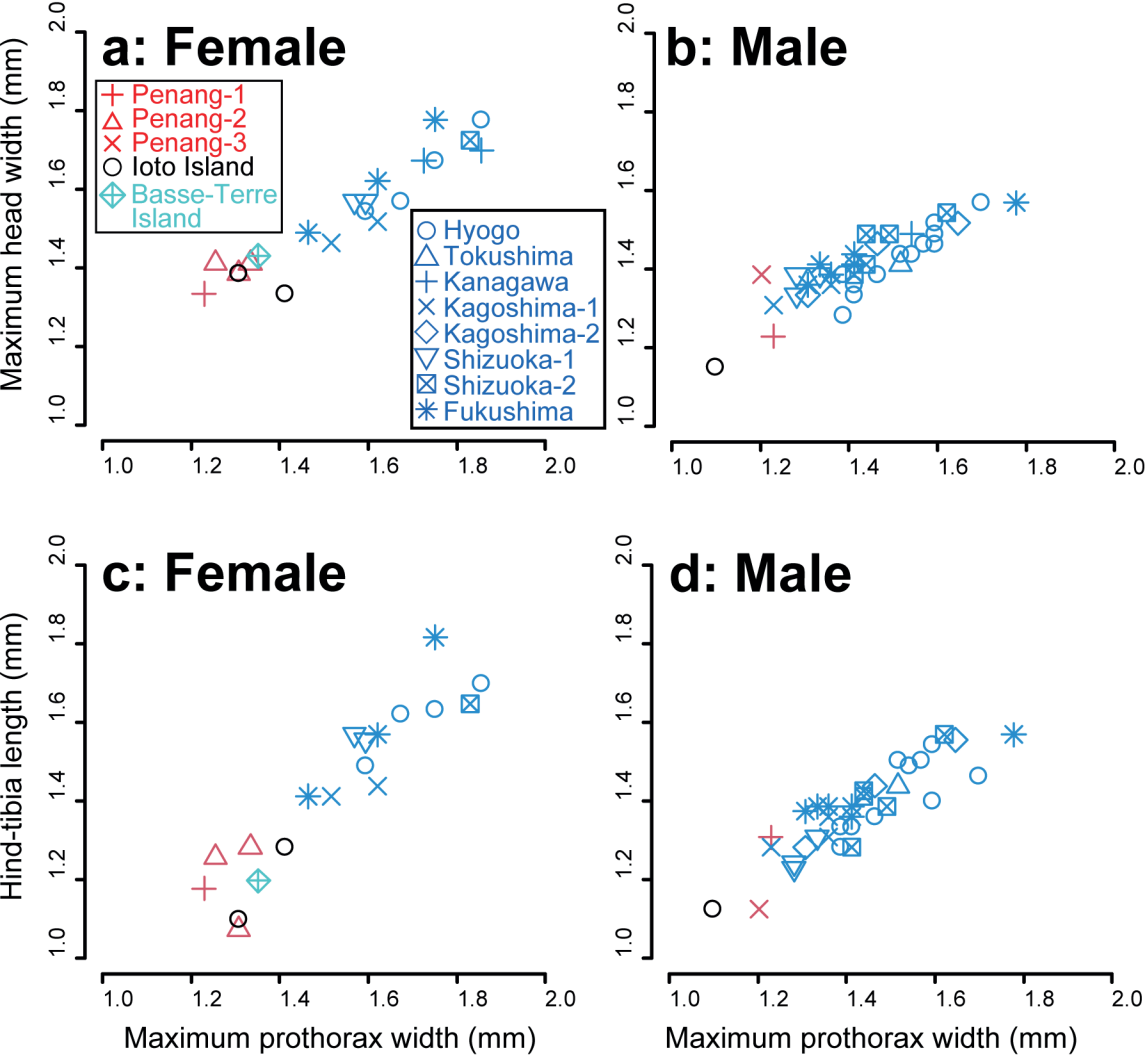
Relationship between the maximum pronotum width and maximum head width (**a, b**), and relationship between the maximum pronotum width and hind tibia length (**c, d**) of female (**a, c**) and male (**b, d**) *Euborellia* species (*Euborellia* sp. 1 and *Euborellia* sp. 3). Red, blue, and black symbols represent samples from Malaysia, the main islands of Japan (Honshu, Shikoku, and Kyushu, and Shimokoshiki Island near Kyushu), or Ioto Island, respectively. The data of a female *E.annulata*, collected from Basse-Terre Island, Guadeloupe Archipelago, is also shown in **a** and **c** (light blue crossed diamonds). Details of the localities are provided in Suppl. material 2.

To separate *Euborellia* sp. 1 and 3, three morphological traits, considered measurable in dried specimens from museums, were quantified and compared: the maximum head width, maximum prothorax width, and hind tibia length. Although the sample size is small for *Euborellia* sp. 1, the three traits were generally smaller in *Euborellia* sp. 1 than in *Euborellia* sp. 3, particularly in females (Fig. 3).

### ﻿Examination of additional materials

**Figure 4.**
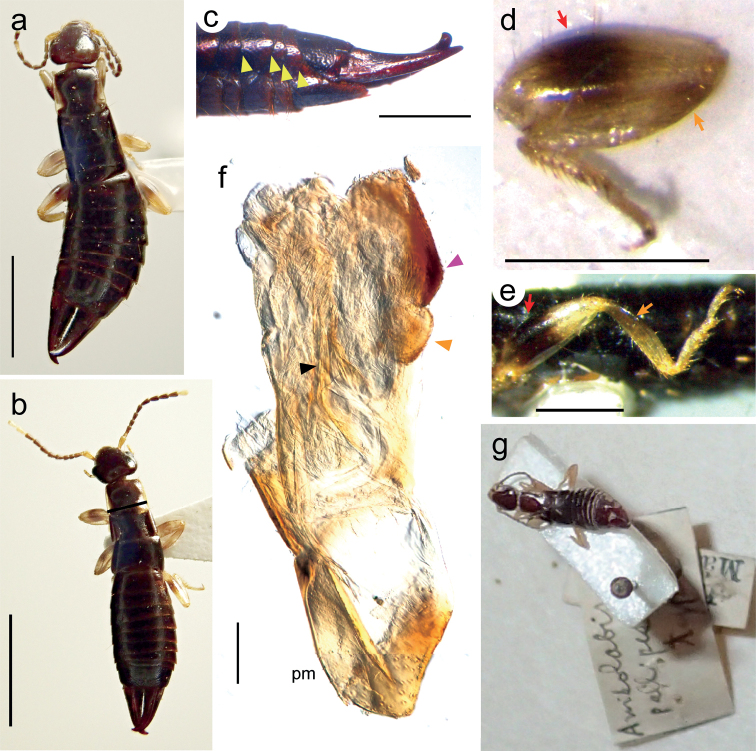
*Euborellia* specimens collected from Ioto Island, Ogasawara Islands, Japan (**a–f**) and the type (holotype) material of *Anisolabispallipes* Shiraki, 1905, preserved in Insect Museum, National Taiwan University, Taipei, Taiwan (**g**). **a** adult female **b** adult male **c** left lateral view of the male post-abdomen **d** right hindleg of the male **e** left hindleg of the female **f** part of the male genitalia (right paramere [pm] and the penis lobe). For the meanings of the arrows and arrowheads, see the caption of Fig. 2. Scale bars: 3 mm (**a, b**); 1 mm (**c–e**); 100 µm (**f**).

As additional materials from Japan, two female and one male adult *Euborellia* collected from Ioto Island (= Iwo-jima Island) in the Ogasawara Islands (= Bonin Islands) were examined. The tegmina of these samples are small flaps (Fig. 4a, b) as in the other *Euborellia* samples examined above. In the male specimen, the lateral side of the 6^th^–9^th^ abdominal tergites protrudes posteriorly and is carinated forming a ridge (Fig. 4c). The conspicuous black band in the femurs (Fig. 4d, e) and the smaller body size compared to *Euborellia* sp. 3 from the main islands of Japan (Fig. 3) indicate that these are identical to *Euborellia* sp. 1. The presence of a Y-shaped area of pigmentation in the male genitalia (Fig. 4f) supports this view. A female sample of *E.annulata* from Basse-Terre Island, Guadeloupe Archipelago (French West Indies), near the type locality, was also placed in the cluster of *Euborellia* sp. 1 based on the morphological measurements (Fig. 3a, c).

Based on a brachypterous adult female collected from Takasago, Hyogo Prefecture, Japan, Shiraki (1905) described *Euborelliapallipes* (Shiraki, 1905) as *Anisolabispallipes* Shiraki, 1905. Although some authors have indicated that the type locality of this species is Taiwan (Formosa) (Burr 1911; Steinmann 1989a, b; Srivastava 2003), the label of the name-bearing type material (female adult), now in the Insect Museum, National Taiwan University, Taipei, Taiwan (Fig. 4g), indicates that it was collected in Takasago (handwritten, in Japanese), Japan (Digital Archives Project of National Taiwan University 2021). This agrees with the original description (Shiraki 1905) and subsequent examination of the type material by Okuni (1913). Although this material has not been examined in detail and morphological measurements have not been made, its leg coloration with pale markings and locality indicate that the specimen belongs to our *Euborellia* sp. 3.

### ﻿DNA barcoding and phylogenetic analysis

**Figure 5.**
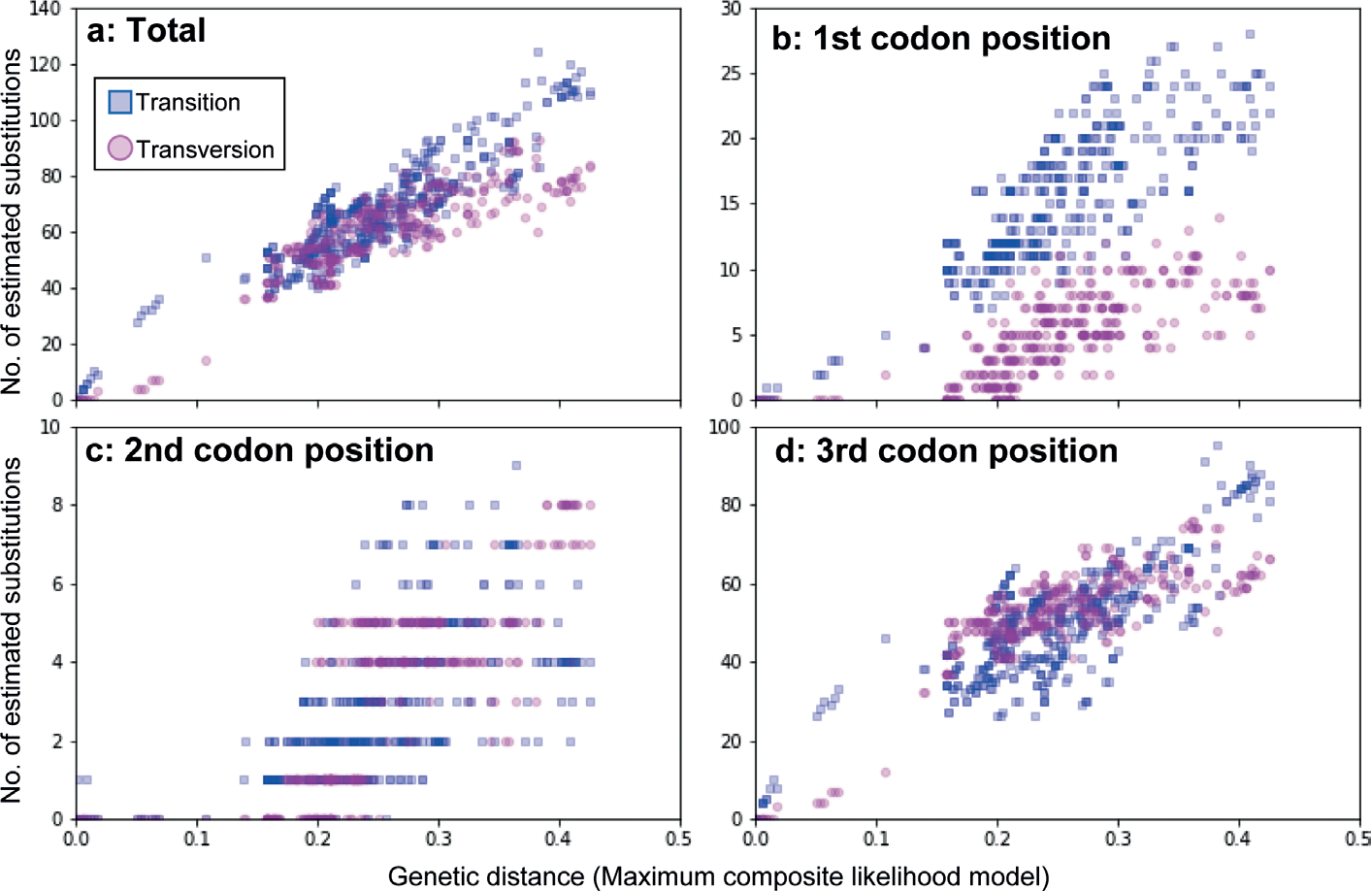
Estimated number of substitutions (transitions and transversions) against the genetic distance (maximum composite likelihood model) in relation to the codon positions.

Although comparable numbers of transversions (Tv) and transitions (Ts) are estimated to occur in the 2^nd^ and 3^rd^ codon positions at the genetic distance larger than ca 0.2 (Fig. 5c, d), in the 1^st^ codon and in total, Ts generally outnumber Tv, both exhibiting a linear relationship with the genetic distance (Fig. 5a, b). Thus, the DNA barcoding region of the Dermaptera is considered to contain phylogenetic information for the diagnoses of species and genera, and relationships among closely related genera.

The percent sequence divergence was lower than 2% within each tentative *Euborellia* species, except for one individual of *Euborellia* sp. 1, which showed about 5% divergence from the other three conspecific samples (Table 1). By contrast, the interspecific divergences were much higher on average: 13.8% between *Euborellia* sp. 1 and 2, 17.0% between *Euborellia* sp. 1 and 3, and 13.7% between *Euborellia* sp. 2 and sp. 3.

**Table 1.** Percent divergence (p-distance) between the sequences. Intraspecific comparisons are highlighted in a different color for each species.

Species (GenBank Accession No.)		1	2	3	4	5	6	7	8	9	10	11	12	13	14	15	16	17
1	*Euborellia* sp. 1 (LC715987)*	0.000																
2	*Euborellia* sp. 1 (LC715988)*	0.055	0.000															
3	*Euborellia* sp. 1 (LC715989)*	0.052	0.015	0.000														
4	*Euborellia* sp. 1 (LC715990)*	0.048	0.012	0.006	0.000													
5	*Euborelliaannulata* (LC740580)	0.018	0.065	0.062	0.059	0.000												
6	*Euborellia* sp. 2 (LC715959)**	0.141	0.139	0.135	0.135	0.142	0.000											
7	*Euborellia* sp. 2 (LC715979)**	0.141	0.139	0.135	0.135	0.142	0.000	0.000										
8	*Euborelliaplebeja* (MW703670.1)	0.153	0.155	0.152	0.150	0.146	0.097	0.097	0.000									
9	*Euborellia* sp. (winged species:MW703671.1)	0.144	0.151	0.150	0.150	0.142	0.109	0.109	0.087	0.000								
10	*Euborellia* sp. 3 (LC715955)***	0.165	0.174	0.167	0.171	0.173	0.136	0.136	0.160	0.153	0.000							
11	*Euborellia* sp. 3 (LC715956)***	0.165	0.174	0.167	0.171	0.173	0.136	0.136	0.160	0.153	0.000	0.000						
12	*Euborellia* sp. 3 (LC715957)***	0.165	0.174	0.167	0.171	0.173	0.136	0.136	0.160	0.153	0.000	0.000	0.000					
13	*Euborellia* sp. 3 (LC715958)***	0.168	0.177	0.170	0.174	0.173	0.139	0.139	0.158	0.154	0.006	0.006	0.006	0.000				
14	*Euborelliafemoralis* (MW703672.1)	0.159	0.152	0.150	0.149	0.155	0.126	0.126	0.150	0.145	0.114	0.114	0.114	0.114	0.000			
15	*Euborelliafemoralis* (MW703673.1)	0.165	0.155	0.152	0.151	0.160	0.124	0.124	0.149	0.147	0.111	0.111	0.111	0.111	0.005	0.000		
16	*Euborelliaannulipes* (LC731318)	0.173	0.174	0.169	0.171	0.171	0.156	0.156	0.152	0.153	0.182	0.182	0.182	0.178	0.166	0.164	0.000	
17	*Euborelliaarcanum* (KP019208.2)	0.171	0.174	0.168	0.168	0.168	0.142	0.142	0.167	0.161	0.188	0.188	0.188	0.192	0.165	0.166	0.179	0.000

* Proposed name: *Euborelliaannulata*. ** Proposed name: *Euborelliaphilippinensis*. *** Proposed name: *Euborelliapallipes*.

**Figure 6.**
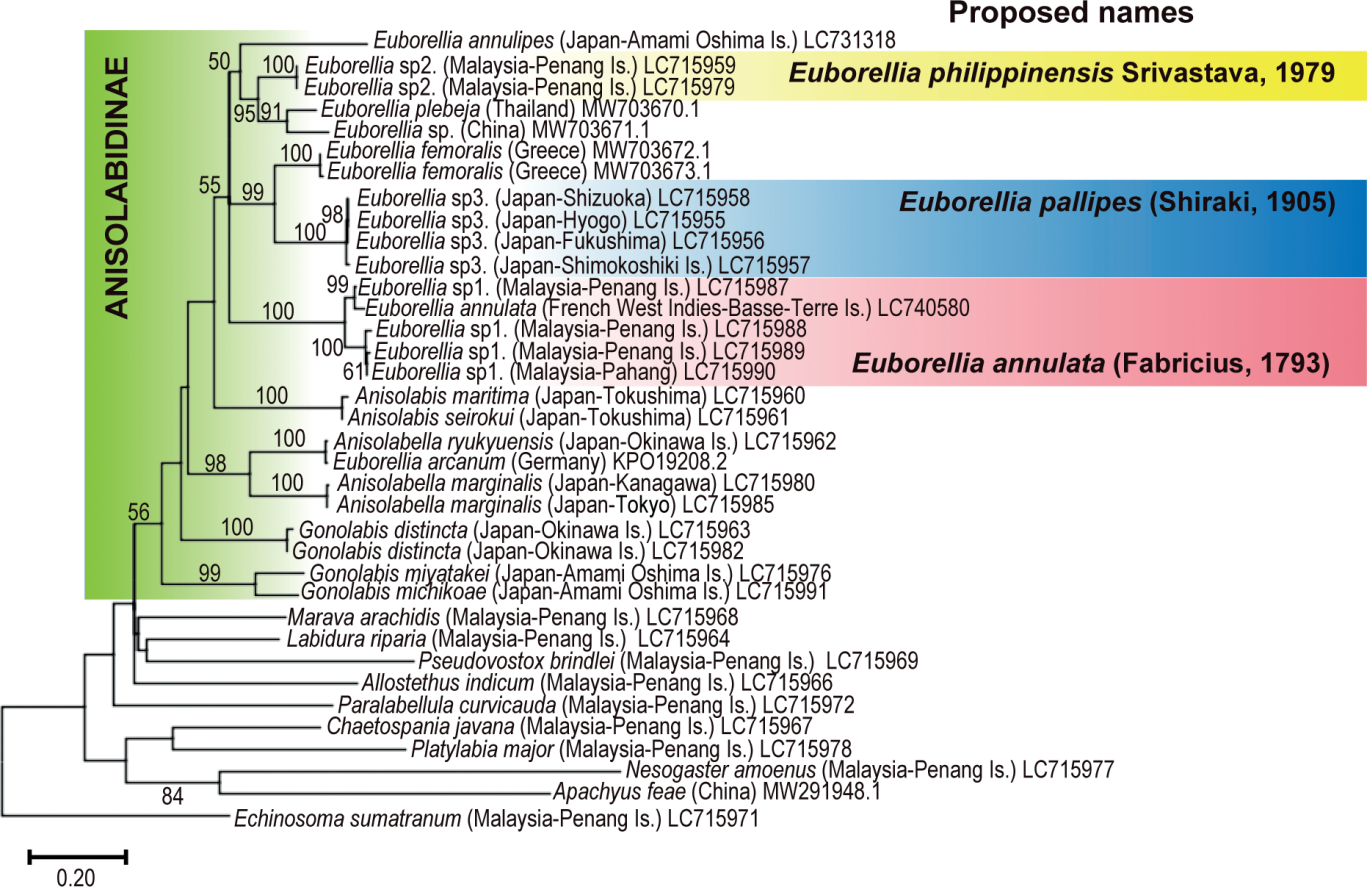
The maximum likelihood phylogenetic tree constructed from COI sequence data. Numbers at the branches indicate bootstrap values (% in 1000 replicates). GenBank accession numbers follow the localities in the parentheses. *Euborellia* sp. 1, *Euborellia* sp. 2, and *Euborellia* sp. 3 are shaded in red, yellow, and blue, respectively, indicating the proposed names. The details of the samples sequenced in the present study (the accession numbers beginning with “LC”), are available in Suppl. material 3 and DDBJ/ENA/GenBank.

Although the support is low (56%), the samples of Anisolabidinae (Anisolabididae) formed a monophyletic clade (Fig. 6). An exception in Anisolabididae is *Platylabiamajor* Dohrn, 1867 (Platylabiinae: = Palicinae Engel & Haas, 2007; = Palexinae Kočárek, 2010), the phylogenetic placement of which was not resolved in our analysis. In Anisolabidinae, *Euborellia* species, except for *Euborelliaarcanum* Matzke & Kočárek, 2015, formed a monophyletic clade (55% support). The DNA barcode region of *E.arcanum*, possibly an introduced species in Europe, is almost identical to that of *Anisolabellaryukyuensis* (Nishikawa, 1969). These species are also similar in the external and genital morphologies (Nishikawa 1969; Matzke and Kočárek 2015), warranting further studies to settle their placements.

In the *Euborellia* clade, multiple samples of each tentative species (*Euborellia* sp. 1, 2, or 3) and *E.femoralis* form monophyletic clades with 100% support. Interestingly, the clade of *Euborellia* sp.1 (from Malaysia) consisted of two subclades, one of which also included *E.annulata* from the West Indies. The sister relationship between *Euborellia* sp. 3 and *E.femoralis* was also supported with high confidence (99%). *Euborellia* sp. (China) and *Euborelliaplebeja* (Dohrn, 1863), for which only the fully winged form has been reported (except for records of those treated as *Euborellia* sp. 3 here), form a clade with 91% support, with its sister place being *Euborellia* sp. 2 (95% support). Placement of *E.annulipes* in this genus was not settled.

### ﻿Identity of the *Euborellia* species

After the description of *Forficulaannulata* from “Americae meridionalis” (= southern America) by Fabricius (1793), the taxonomy of *Euborellia* species with flap-like, abbreviated tegmina has long been confused. De Bormans and Krauss (1900) and Burr (1911) listed this species as *Labiaannulata* under Spongiphoridae (= Labiidae), and Steinmann (1989b, 1990) followed this view. Brindle (1981), who examined the Fabrician types in the Zoological Museum of Copenhagen University, concluded that the type of *Forficulaannulata*, a male collected from the West Indies, is identical to the species known as *Euborelliastali* (Dohrn, 1984) (see also Kevan and Vickery 1997). Accordingly, many subsequent authors treated *E.stali* as a junior synonym of the circumtropical cosmopolitan species *E.annulata*, together with *Anisolabisminuta* Caudell, 1907 (the type locality is Puerto Rico), which Hebard (1923) and Reichardt (1968) proposed to be a junior synonym of *E.stali* (Sakai 1987; Srivastava 2003).

Interestingly, our *Euborellia* sp. 1 made a cluster, both in morphology (Fig. 3) and molecular (Fig. 6) data sets, with the female sample of *E.annulata* collected near its type locality. Thus, we follow the view that *E.annulata* is a circumtropical cosmopolitan, and assign our *Euborellia* sp. 1 to *E.annulata*, as we did in Kamimura et al. (2016). In the present study, we detected the characteristic Y-shaped area of pigmentation on the penis lobe of *Euborellia* sp. 1. Unfortunately, previous descriptions of *Euborellia* species, including those of *E.annulata*, *E.stali*, and *E.minuta*, do not include detailed structures on the penis lobes. Extensive re-examination for this trait is required for the type material and specimens assigned as *E.annulata* from other localities. Our examination of the samples from Ioto Island in the Ogasawara Islands, particularly the detection of a Y-shaped area of pigmentation on the penis lobe of the male specimen, indicates that they are conspecific to our *Euborellia* sp. 1, and thus *E.annulata* (Figs 3, 4a–f). Brindle (1972) reported the occurrence of *E.annulata* (as *E.stali*) in the Pacific, including Chichijima Island in the Ogasawara Islands, which is approximately 200 km north of Ioto Island (see also Nishikawa 2020b).

Interestingly, our molecular analysis detected two sub-clades in *Euborelliaannulata*: a female from Bayan Lepas, Penang Island, Malaysia (LC715987) showed only 1.8% divergence from a female from the West Indies (LC740580), while the other Malaysian samples (LC715988–LC715990), including those from other sites of Penang Island, were clustered with 4.8–6.5% differences from the formers (Table 1). We detected no differences in the external morphology between these two subgroups. Considering that some widely-distributed insects show remarkable intraspecific diversity (> 5%) in the CO1 sequences (up to 26.0%: Cognato 2006), we tentatively treat those as a single species *E.annulata*. Future studies should explore for possible isolations among possible sub-lineages of this species.

Kamimura et al. (2016) treated *Euborellia* sp. 2 as *E.philippinensis*, although no fully winged morph had been reported for this species. The external and male genital morphology of the brachypterous form of *Euborellia* sp. 2 agree with the previous descriptions of *E.philippinensis* (Srivastava 1979), including the sharp external apical angle of the parameres (Fig. 2r: black arrows) and the posterior margin and angles of the pronotum broadly rounded (Fig. 2b), compared to those of *E.annulata*.

In addition to *Euborellia* sp. 2, two macropterous *Euborellia* species, *E.plebeja* and *E.femoralis*, have been reported from the Orient (Srivastava 2003). Although the treatment of *E.stali* (with abbreviated tegmina) as a junior synonym of *E.plebeja* by Hebard (1927) caused further confusion and difficulties in the taxonomy of *Euborellia* (Brindle 1972), except for the erroneous treatments of *Euborellia* sp. 3 discussed below, no indisputable example of an apterous or brachypterous form of *E.plebeja* has not been reported. According to Bey-Bienko (1959) and Srivastava (2003), lateral ridges (carina) do not develop in *E.plebeja* (vs. developed in the 6^th^–9^th^ abdominal tergites of *E.femoralis*) with more prominent external angles of the parameres (vs. external angles convex in *E.femoralis*). Thus, it is difficult to distinguish the macropterous form of *Euborellia* sp. 2, found only in laboratory-reared individuals, from *E.plebeja* (Kamimura et al. 2016). Our phylogenetic analysis also revealed that *Euborellia* sp. 2 is closely related to *E.plebeja* (a Thailand specimen) and *Euborellia* sp. (a Chinese specimen; macropterous), both of which Kalaentzis et al. (2021) sequenced. However, the genetic differentiation in the DNA barcode region is relatively large between *Euborellia* sp. 2 and *E.plebeja* (more than 9.5%: Fig. 6). Kalaentzis et al. (2021) considered that *Euborellia* sp. from China, which is sister to *E.plebeja* with genetic differentiation of about 8.7%, represents another species. Following this view, we tentatively treat *Euborellia* sp. 2 as *E.philippinensis* based on the morphology of the brachypterous form, as we did in Kamimura et al. (2016).

The present results clearly show that *Euborellia* sp. 3 of the main islands of Japan (Honshu, Shikoku, and Kyushu) is a distinct species. After the proposed (and erroneous) synonymy of *E.minuta* and *E.stali* as *E.plebeja* (Hebard 1923, 1927), Hebard (1933) also treated *Anisolabispallipes* Shiraki, 1905 as a junior synonym of *E.plebeja*. Steinmann (1989a, b) proposed the same synonymy for *A.pallipes*. By contrast, Srivastava (2003) considered *A.pallipes* a junior synonym of *E.annulata* (= *E.stali*). Accordingly, the names *E.plebeja*, *E.stali*, or *E.annulata* have been used for the brachypterous *Euborellia* recorded from the main islands of Japan. However, judging from the type locality and external appearance of the type material bearing this name, we resurrect the name *Euborelliapallipes* (Shiraki, 1905) for our *Euborellia* sp. 3. Thus, though many subsequent authors treated (or suggested treating) this species, which closely resembles our *Euborellia* sp. 1, as a junior synonym of *E.annulata* (Srivastava 2003; Nishikawa 2011, 2016, 2020a), *E.stali* (Nishikawa 1975), or the macropterous species *E.plebeja* (Hincks 1947; Nishikawa 1969; Steinmann 1989a, b; Chen and Ma 2004), we consider that our *Euborellia* sp. 3 is *E.pallipes*, which is distinct from the above-mentioned species. Our identification and diagnoses are summarized in Table 2.

**Table 2.** Diagnostic features of the three brachypterous *Euborellia* species from East and Southeast Asia. Female *E.annulata* and *E.pallipes* are difficult to distinguish, but the former is usually smaller (Fig. 3).

Traits	*Euborelliaannulata* (*Euborellia* sp. 1)	*Euborelliaphilippinensis* (*Euborellia* sp. 2)	*Euborelliapallipes* (*Euborellia* sp. 3)
Black markings of legs	Markings of mid femur are darker than those of basal half of tibia	Markings of mid femur are weaker than those of basal half of tibia	In almost same intensity
Lateral sides of male abdominal segments 6^th^ (or 7^th^) to 9^th^	Carinated	Not carinated	Carinated
Outer margin of parameres	Not strongly angular, rounded	Strongly angular	Not strongly angular, rounded
Y-shaped area of pigmentation on penis lobes	Present	Absent	Absent

Interestingly, our molecular analysis revealed that this species is sister to *E.femoralis* (Fig. 6), for which only totally apterous or fully winged individuals have been reported (Bey-Bienko 1959; Anisyutkin 1998; Kalaentzis et al. 2021). Although Steinmann (1989a, b) treated the brachypterous species *Anisolabisminuta* Caudell, 1907 (= *E.minuta*) as a junior system of *E.femoralis*, this treatment lacks foundation (Srivastava 2003). The identities of *Euborellia* samples with abbreviated tegmina from Taiwan, the Nansei Islands of Japan, Korea, and mainland China, reported under the names *E.annulata* (Nishikawa 2016), *E.pallipes* (Shiraki 1928; Bey-Bienko 1936, 1959; Masaki 1936; Cho 1969; Sakai 1970, 1982; Moon and Kim 1983; Kim and Moon 1985), or *E.plebeja* (Moon and Kim 1983, 1991; Kim and Moon 1985; Sakai 1987; Chen and Ma 2004), are not determined at present. Some other brachypterous *Euborellia* species have also been reported from South Asia to the Middle East: *E.abbreviata* Srivastava, 1977 [India], *E.annandalei* (Burr, 1906) [India], *E.manipurensis* Srivastava, 1979 [India], *E.sakaii* Steinmann, 1978 [Afghanistan], and *E.moesta* Géné, 1839 [Iran] (Srivastava 2003; Kočárek 2011a, b), relationships of which to the species studied here are totally unclear. Although the present study shows the effectiveness of DNA barcoding for specific diagnoses of *Euborellia* species, only limited entries are available for the Dermaptera in the sequence data banks. Examinations of molecular and morphological data are required for additional materials, as well as rearing experiments for investigating wing polymorphisms.

## Supplementary materials

Supplementary material 1 The samples and results of the crossing experiments Data type table (Pdf file) https://binary.pensoft.net/file/806533This dataset is made available under the Open Database License (http://opendatacommons.org/licenses/odbl/1.0/). The Open Database License (ODbL) is a license agreement intended to allow users to freely share, modify, and use this Dataset while maintaining this same freedom for others, provided that the original source and author(s) are credited.Yoshitaka Kamimura, Chow-Yang Lee, Junsuke Yamasako, Masaru Nishikawa

Supplementary material 2 The specimens and results of morphological measurements Data type table (Pdf file) https://binary.pensoft.net/file/806534This dataset is made available under the Open Database License (http://opendatacommons.org/licenses/odbl/1.0/). The Open Database License (ODbL) is a license agreement intended to allow users to freely share, modify, and use this Dataset while maintaining this same freedom for others, provided that the original source and author(s) are credited.Yoshitaka Kamimura, Chow-Yang Lee, Junsuke Yamasako, Masaru Nishikawa

Supplementary material 3 Species and the collection sites of the Dermaptera samples sequenced in this study Data type table (Pdf file) https://binary.pensoft.net/file/806535This dataset is made available under the Open Database License (http://opendatacommons.org/licenses/odbl/1.0/). The Open Database License (ODbL) is a license agreement intended to allow users to freely share, modify, and use this Dataset while maintaining this same freedom for others, provided that the original source and author(s) are credited.Yoshitaka Kamimura, Chow-Yang Lee, Junsuke Yamasako, Masaru Nishikawa

## References

[B1] Anisyutkin LN (1998) To the knowledge of earwigs of the subfamily Anisolabidinae (Dermaptera, Anisolabididae) from SE Asia. Entomological Review 78: 627–641.

[B2] Bey-Bienko GY (1936) Nasekomye kochistokrylye (Dermaptera). Fauna SSSR 5. [Earwigs (Dermaptera). Fauna USSR 5]. Izdatelstvo Akademii Nauk USSR, Moskva – Leningrad, 239 pp. https://djvu.online/file/FUxNgqgCVapfz [In Russian]

[B3] Bey-Bienko GY (1959) Dermaptera of Sichuan and Yunnan. Results of Chinese-Soviet Zoological and Botanical Expeditions to SW China, 1955–1957. Entomologicheskoe Obozrenie 38: 590–672. [In Russian]

[B4] Brindle A (1972) Dermaptera. Insects of Micronesia 5: 97–171.

[B5] Brindle A (1981) The types of Dermaptera described by Fabricius. Entomologist’s Record and Journal of Variation 93: 14–16.

[B6] Burr M (1911) Dermaptera. Genera Insectorum, Bruxelles 122: 1–112. 10.5962/bhl.part.9310

[B7] Chen Y Ma W (2004) Dermaptera. Fauna Sinica 35: 1–420. [In Chinese]

[B8] Cho PS (1969) Dermaptera. Illustrated Encyclopedia of Fauna & Flora of Korea (Vol. 10). Insecta (II), Mun-gyobu, Seoul, 802–816 pp. [pl. 55] [In Korean]

[B9] Cognato AI (2006) Standard percent DNA sequence difference for insects does not predict species boundaries. Journal of Economic Entomology 99(4): 1037–1045. 10.1093/jee/99.4.1037 16937653

[B10] de Bormans A Krauss H (1900) Forficulidae und Hemimeridae – Das Tierreich 11. Verlag von R. Friedländer und Sohn, Berlin, 142 pp. 10.5962/bhl.title.69336

[B11] Digital Archives Project of National Taiwan University (2021) Digital Archives Project of National Taiwan University. http://www.darc.ntu.edu.tw/newdarc/darc/english/introduction.htm [Accessed on 2022-7-1]

[B12] Dohrn H (1864) Versuch einer Monographie der Dermapteren. Stettin entomologische Zeitung 25: 285–296.

[B13] Engel MS Haas F (2007) Family-group names for earwigs (Dermaptera). American Museum Novitates 3567(1): 1–20. 10.1206/0003-0082(2007)539[1:FNFED]2.0.CO;2

[B14] Fabricius JC (1793) Entomologia Systematica Emendata et Aucta. Secundum Classes, Ordines, Genera, Species Adjectis Synonimis, Locis, Observationibus, Descriptionibus. Tome 2. Impensis Christ. Gottl. Proft, Hafniae, 519 pp. 10.5962/bhl.title.122153

[B15] Folmer O Black M Hoeh W Lutz R Vrijenhoek R (1994) DNA primers for amplification of mitochondrial cytochrome c oxidase subunit I from diverse metazoan invertebrates. Molecular Marine Biology and Biotechnology 3: 294–299. 10.1071/ZO9660275 7881515

[B16] Hadley A (2008) Combine ZM imaging software. https://combinezm.en.lo4d.com/details [Accessed on 2018-2-10]

[B17] Hebard M (1923) Studies in Indian Dermaptera. Memoirs of the Department of Agriculture in India, Entomological Series 7: 195–242.

[B18] Hebard M (1927) Studies in Sumatran Dermaptera. Proceedings. Academy of Natural Sciences of Philadelphia 79: 23–48.

[B19] Hebard M (1933) Dermaptera in the collection of the California Academy of Sciences. The Pan-Pacific Entomologist 9: 140–144.

[B20] Hincks WD (1947) Preliminary notes on Mauritian earwigs (Dermaptera). Annals & Magazine of Natural History 14(116): 517–540. 10.1080/00222934708654662

[B21] Hopkins H Maehr MD Haas F Deem LS (2018) Dermaptera Species File. Version 5.0/5.0. [WWW document] http://Dermaptera.SpeciesFile.org [Accessed on 2022-7-31]

[B22] Kalaentzis K Kazilas C Agapakis G Kocarek P (2021) Hidden in plain sight: First records of the alien earwig *Euborelliafemoralis* (Dohrn, 1863) in Europe. BioInvasions Records 10(4): 1022–1031. 10.3391/bir.2021.10.4.27

[B23] Kamimura Y Nishikawa M Lee C-Y (2016) The earwig fauna (Insecta: Dermaptera) of Penang Island, Malaysia, with descriptions of two new species. Zootaxa 4084(2): 233–257. 10.11646/zootaxa.4084.2.4 27394261

[B24] Kevan DKMcE Vickery VR (1997) An annotated provisional list of non-saltatorial orthopteroid insects of Micronesia, compiled mainly from the literature. Micronesia 30: 269–353.

[B25] Kim CW Moon TY (1985) A taxonomic revision of Korean Dermaptera. Entomological Research Bulletin [Korea] 11: 37–59.

[B26] Kočárek P (2010) Case 3522. Palicinae Burr, 1910 (Dermaptera, Spongiphoridae): Proposed emendation of the current spelling to Palexinae to remove homonymy with Palicidae Bouvier, 1898 (Crustacea, Decapoda). Bulletin of Zoological Nomenclature 67(3): 211–212. 10.21805/bzn.v67i3.a8

[B27] Kočárek P (2011a) Dermaptera of Iran with description of *Euborelliaangustata* sp. nov. Acta Entomologica Musei Nationalis Pragae 51: 381–390.

[B28] Kočárek P (2011b) *Euborelliaornata* sp. nov. from Nepal (Dermaptera: Anisolabididae). Acta Entomologica Musei Nationalis Pragae 51: 391–395.

[B29] Kočárek P Wahab RA (2021) Termitophily documented in earwigs (Dermaptera). Biology 10: e1243. 10.3390/biology10121243 PMC8698772 34943158

[B30] Masaki J (1936) On the insect-fauna of various islands of Korea (I). Kontyû 10: 251–274. [In Japanese]

[B31] Matzke D Kočárek P (2015) Description and biology of *Euborelliaarcanum* sp. nov., an alien earwig occupying greenhouses in Germany and Austria (Dermaptera: Anisolabididae). Zootaxa 3956(1): 131–139. 10.11646/zootaxa.3956.1.8 26248909

[B32] Moon TY Kim CW (1983) The systematic study of Korean Dermaptera. I. (Carcinophorinae, Carcinophoridae). Entomological Research Bulletin [Korea] 9: 29–42.

[B33] Moon TY Kim CW (1991) Catalogue of Korean Dermaptera. Entomological Research Bulletin [Korea] 17: 67–79.

[B34] Nishikawa M (1969) Notes on the Carcinophorinae of Japan and Ryukyus (Dermaptera: Carcinophoridae). Kontyû 37: 41–55.

[B35] Nishikawa M (1975) Dermaptera. In: Ishihara T (Ed.) Pictorial Encyclopedia of Insect III for Student.Gakken, Tokyo, 65–66. [(plates) and 187–383 pp.] [In Japanese]

[B36] Nishikawa M (2011) Unidentified earwig (Dermaptera) specimens preserved in the Hiwa Museum for Natural History, Shobara City, Hiroshima, Japan, with a check-list of the earwigs of Hiroshima Prefecture. Miscellaneous Reports of the Hiwa Museum for Natural History 52: 1–12. [In Japanese with English abstract]

[B37] Nishikawa M (2016) Dermaptera. In: Orthopterological Society of Japan (Ed.) The Standard of Polyneoptera in Japan. Gakken Plus, Tokyo, 170‒186. [In Japanese]

[B38] Nishikawa M (2020a) Order Dermaptera. In: Editorial Committee of Catalogue of the Insects of Japan (Ed.) Catalogue of the Insects of Japan (Vol. 3): Polyneoptera. Touka Shobo, Fukuoka, Japan, 56‒67. [In Japanese]

[B39] Nishikawa M (2020b) Dermaptera of the Bonin Islands, Japan. Battarigisu 163: 191–196. [In Japanese]

[B40] Okuni T (1913) Verzeichnis der Japanischen Euplexopteren. Transactions of the Sapporo Natural History Society 4: 182‒188. https://eprints.lib.hokudai.ac.jp/dspace/handle/2115/60865

[B41] Popham EJ Brindle A (1966) Genera and species of the Dermaptera. 3. Carcinophorinae and Arixeniidae. Entomologist, London 99: 269–278.

[B42] Reichardt H (1968) Catalogue of New World Dermaptera (Insecta). Part II. Labioides, Carcinophoridae. Papéis Avulsos de Zoologia [São Paulo] 22: 35–46.

[B43] Sakai S (1970) Dermapterorum Catalogus Praeliminaris. I. Labiduridae and Carcinophoridae. Daito Bunka University, Tokyo, 49 pp. [and 91 pp.]

[B44] Sakai S (1982) A new proposed classification of the Dermaptera with special reference to the check-list of the Dermaptera of the world. Bulletin of Daito Bunka University 20: 1–108.

[B45] Sakai S (1987) Dermapterorum Catalogus XIX–XX: Iconographia IV–V. Chelisochidae and Anisolabididae. Daito Bunka University, Tokyo, 1567 pp.

[B46] Shiraki T (1905) Neue Forficuliden Japans. Transactions of the Sapporo Natural History Society 1: 91–96. http://hdl.handle.net/2115/60754

[B47] Shiraki T (1928) Dermapteren aus dem Kaiserreich Japan. Insecta Matsumurana 3: 1–25. http://hdl.handle.net/2115/9156

[B48] Srivastava GK (1979) On a new species of genus *Euborellia* Burr (Dermaptera: Carcinophoridae) from Philippines. Bulletin of the Zoological Survey of India 2: 49–51.

[B49] Srivastava GK (1999) On the higher classification of Anisolabididae (Insecta: Dermaptera) with a check-list of genera and species. Records of the Zoological Survey of India 97(1): 73–100. 10.26515/rzsi/v97/i1/1999/160257

[B50] Srivastava GK (2003) Fauna of India and Adjacent Countries, Dermaptera Part II: Anisolabidoidea. Zoological Survey of India, Kolkata, India, 235 pp.

[B51] Steinmann H (1989a) Dermaptera. Catadermaptera II. Das Tierreich 105: 1–504. 10.1515/9783112419687

[B52] Steinmann H (1989b) World Catalogue of Dermaptera. Kluwer Academic Publishers, Dordrecht, Netherlands, 934 pp.

[B53] Steinmann H (1990) Dermaptera. Eudermaptera I. Das Tierreich 106: 1–558.

[B54] Stuart OP Binns M Umina PA Holloway J Severtson D Nash M Heddle T van Helden M Hoffmann AA (2019) Morphological and molecular analysis of australian earwigs (Dermaptera) points to unique species and regional endemism in the Anisolabididae family. Insects 10(3): 1–25. 10.3390/insects10030072 PMC6468374 30875825

[B55] Su ZH Imura Y Okamoto M Osawa S (2004) Pattern of phylogenetic diversification of the Cychrini ground beetles in the world as deduced mainly from sequence comparisons of the mitochondrial genes. Gene 326: 43–57. 10.1016/j.gene.2003.10.025 14729262

[B56] Tamura K Stecher G Kumar S (2021) MEGA11: Molecular Evolutionary Genetics Analysis version 11. Molecular Biology and Evolution 38(7): 3022–3027. 10.1093/molbev/msab120 33892491 PMC8233496

[B57] Thompson JD Gibson TJ Higgins DG (2003) Multiple sequence alignment using ClustalW and ClustalX. Current Protocols in Bioinformatics 00(1): 2–3. 10.1002/0471250953.bi0203s00 18792934

[B58] Zhang ZQ (2013) Phylum Arthropoda. In: Zhang Z-Q (Ed.) Animal Biodiversity: An outline of higher-level classification and survey of taxonomic richness (Addenda 2013).Zootaxa 3703: 17–26. 10.11646/zootaxa.3703.1.6 26146682

