# Supplementary material for: ﻿Identification and reproductive isolation of Euborellia species (Insecta, Dermaptera, Anisolabididae) from East and Southeast Asia
Source: Zookeys. 2023 Feb 7;1146:115–34. doi: 10.3897/zookeys.1146.98248 (PMC10194414; doi:10.3897/zookeys.1146.98248)
Supplement: Supplementary material 1 — The samples and results of the crossing experiments [file zookeys-1146-115_article-98248__-s001.pdf]

Table S1. The samples and results of the crossing experiments.

Experiment No.	Cross type (female species x male species)	Female age	Male age	Insemination*	Oviposition**	Hatchlings production***	Female origin (population-line)	Male origin (population-line)
Exp. 1	sp. 1 x sp. 1	11	Wild-caught	1	NA	NA	MALAYSIA (Penang Island, Bukit Bendera)	MALAYSIA(Pahang, Kuantan)
Exp. 1	sp. 1 x sp. 1	6	Wild-caught	1	NA	NA	MALAYSIA (Penang Island, Bukit Bendera x Pahang, Kuantan)	MALAYSIA (Penang Island, Bayan Lepas)
Exp. 1	sp. 1 x sp. 1	6	24	1	NA	NA	MALAYSIA (Penang Island, Batu Ferringi x Penang Island, Bukit Bendera)	MALAYSIA (Penang Island, Bukit Bendera x Pahang, Kuantan)
Exp. 1	sp. 1 x sp. 1	6	15	1	NA	NA	MALAYSIA (Penang Island, Batu Ferringi)	MALAYSIA (Penang Island, Batu Ferringi x Penang Island, Bukit Bendera)
Exp. 1	sp. 1 x sp. 1	6	Wild-caught	1	NA	NA	MALAYSIA (Penang Island, Bayan Lepas)	MALAYSIA (Penang Island, Bayan Lepas)
Exp. 1	sp. 1 x sp. 2	11	11	1	NA	NA	MALAYSIA (Penang Island, Bukit Bendera)	MALAYSIA (Penang Island, Sungai Nipah)
Exp. 1	sp. 1 x sp. 2	6	21	1	NA	NA	MALAYSIA (Penang Island, Bukit Bendera x Pahang, Kuantan)	MALAYSIA (Penang Island, Sungai Nipah)
Exp. 1	sp. 1 x sp. 2	6	51	1	NA	NA	MALAYSIA (Penang Island, Batu Ferringi x Penang Island, Bukit Bendera)	MALAYSIA (Penang Island, Sungai Nipah)
Exp. 1	sp. 1 x sp. 2	6	15	1	NA	NA	MALAYSIA (Penang Island, Bayan Lepas)	MALAYSIA (Penang Island, Sungai Nipah)
Exp. 1	sp. 1 x sp. 2	9	6	0	NA	NA	MALAYSIA (Penang Island, Bayan Lepas)	MALAYSIA (Penang Island, Sungai Nipah)
Exp. 1	sp. 1 x sp. 3	12	15	0	NA	NA	MALAYSIA (Penang Island, Bukit Bendera x Pahang, Kuantan)	JAPAN (Tokushima, Tokushima)
Exp. 1	sp. 1 x sp. 3	6	6	1	NA	NA	MALAYSIA (Penang Island, Batu Ferringi x Penang Island, Bukit Bendera)	JAPAN (Tokushima, Tokushima)
Exp. 1	sp. 1 x sp. 3	6	9	0	NA	NA	MALAYSIA (Penang Island, Bayan Lepas)	JAPAN (Tokushima, Tokushima)
Exp. 1	sp. 1 x sp. 3	6	9	0	NA	NA	MALAYSIA (Penang Island, Bayan Lepas)	JAPAN (Tokushima, Tokushima)
Exp. 1	sp. 1 x sp. 3	68	12	0	NA	NA	MALAYSIA (Penang Island, Bayan Lepas)	JAPAN (Kanagawa, Yokohama)
Exp. 1	sp. 2 x sp. 1	17	17	0	NA	NA	MALAYSIA (Penang Island, Sungai Nipah)	MALAYSIA (Penang Island, Bukit Bendera)
Exp. 1	sp. 2 x sp. 1	57	Wild-caught	0	NA	NA	MALAYSIA (Penang Island, Sungai Nipah)	MALAYSIA(Penang Island, Batu Ferringi)
Exp. 1	sp. 2 x sp. 1	9	Wild-caught	0	NA	NA	MALAYSIA (Penang Island, Sungai Nipah)	MALAYSIA (Penang Island, Bayan Lepas)
Exp. 1	sp. 2 x sp. 1	6	Wild-caught	0	NA	NA	MALAYSIA (Penang Island, Sungai Nipah)	MALAYSIA (Penang Island, Bayan Lepas)
Exp. 1	sp. 2 x sp. 1	6	Wild-caught	0	NA	NA	MALAYSIA (Penang Island, Sungai Nipah)	MALAYSIA(Penang Island, Batu Ferringi)
Exp. 1	sp. 2 x sp. 2	9	6	1	NA	NA	MALAYSIA (Penang Island, Sungai Nipah)	MALAYSIA (Penang Island, Sungai Nipah)
Exp. 1	sp. 2 x sp. 2	6	9	1	NA	NA	MALAYSIA (Penang Island, Sungai Nipah)	MALAYSIA (Penang Island, Sungai Nipah)
Exp. 1	sp. 2 x sp. 2	12	21	1	NA	NA	MALAYSIA (Penang Island, Sungai Nipah)	MALAYSIA (Penang Island, Sungai Nipah)
Exp. 1	sp. 2 x sp. 2	6	33	1	NA	NA	MALAYSIA (Penang Island, Sungai Nipah)	MALAYSIA (Penang Island, Sungai Nipah)
Exp. 1	sp. 2 x sp. 2	19	6	1	NA	NA	MALAYSIA (Penang Island, Sungai Nipah)	MALAYSIA (Penang Island, Sungai Nipah)
Exp. 1	sp. 2 x sp. 3	57	Wild-caught	0	NA	NA	MALAYSIA (Penang Island, Sungai Nipah)	JAPAN (Tokushima, Tokushima)
Exp. 1	sp. 2 x sp. 3	24	6	0	NA	NA	MALAYSIA (Penang Island, Sungai Nipah)	JAPAN (Tokushima, Tokushima)
Exp. 1	sp. 2 x sp. 3	34	22	1	NA	NA	MALAYSIA (Penang Island, Sungai Nipah)	JAPAN (Tokushima, Tokushima)
Exp. 1	sp. 2 x sp. 3	19	12	0	NA	NA	MALAYSIA (Penang Island, Sungai Nipah)	JAPAN (Tokushima, Tokushima)
Exp. 1	sp. 2 x sp. 3	6	35	1	NA	NA	MALAYSIA (Penang Island, Sungai Nipah)	JAPAN (Tokushima, Tokushima)
Exp. 1	sp. 3 x sp. 1	6	24	1	NA	NA	JAPAN (Tokushima, Tokushima)	MALAYSIA (Penang Island, Bukit Bendera x Pahang, Kuantan)
Exp. 1	sp. 3 x sp. 1	9	9	1	NA	NA	JAPAN (Tokushima, Tokushima)	MALAYSIA (Penang Island, Bayan Lepas)
Exp. 1	sp. 3 x sp. 1	6	9	1	NA	NA	JAPAN (Tokushima, Tokushima)	MALAYSIA (Penang Island, Bayan Lepas)
Exp. 1	sp. 3 x sp. 1	15	68	1	NA	NA	JAPAN (Kanagawa, Yokohama)	MALAYSIA (Penang Island, Bayan Lepas)
Exp. 1	sp. 3 x sp. 1	51	32	0	NA	NA	JAPAN (Kanagawa, Yokohama)	MALAYSIA (Penang Island, Bayan Lepas)
Exp. 1	sp. 3 x sp. 2	9	48	0	NA	NA	JAPAN (Tokushima, Tokushima)	MALAYSIA (Penang Island, Sungai Nipah)
Exp. 1	sp. 3 x sp. 2	5	14	1	NA	NA	JAPAN (Tokushima, Tokushima)	MALAYSIA (Penang Island, Sungai Nipah)
Exp. 1	sp. 3 x sp. 2	9	Wild-caught	0	NA	NA	JAPAN (Tokushima, Tokushima)	MALAYSIA (Penang Island, Sungai Nipah)
Exp. 1	sp. 3 x sp. 2	15	45	0	NA	NA	JAPAN (Kanagawa, Yokohama)	MALAYSIA (Penang Island, Sungai Nipah)
Exp. 1	sp. 3 x sp. 2	11	14	0	NA	NA	JAPAN (Kanagawa, Yokohama)	MALAYSIA (Penang Island, Sungai Nipah)
Exp. 1	sp. 3 x sp. 3	5	23	1	NA	NA	JAPAN (Tokushima, Tokushima)	JAPAN (Tokushima, Tokushima)
Exp. 1	sp. 3 x sp. 3	9	6	1	NA	NA	JAPAN (Tokushima, Tokushima)	JAPAN (Tokushima, Tokushima)
Exp. 1	sp. 3 x sp. 3	46	79	1	NA	NA	JAPAN (Kanagawa, Yokohama)	JAPAN (Tokyo, Komae)
Exp. 1	sp. 3 x sp. 3	6	Wild-caught	1	NA	NA	JAPAN (Kanagawa, Yokohama)	JAPAN (Kanagawa, Yokohama)
Exp. 1	sp. 3 x sp. 3	8	8	1	NA	NA	JAPAN (Kanagawa, Yokohama)	JAPAN (Kanagawa, Yokohama)
Exp. 2	sp. 1 x sp. 1	8	Wild-caught	1	1	1	MALAYSIA (Penang Island, Bukit Bendera)	MALAYSIA(Pahang, Kuantan)
Exp. 2	sp. 1 x sp. 1	10	3	1	1	1	MALAYSIA (Penang Island, Bayan Indah beach)	MALAYSIA (Penang Island, Bukit Bendera)
Exp. 2	sp. 1 x sp. 1	9	27	1	1	1	MALAYSIA (Penang Island, Bayan Lepas)	MALAYSIA (Penang Island, Bayan Lepas)
Exp. 2	sp. 1 x sp. 1	9	Wild-caught	1	1	1	MALAYSIA (Penang Island, Batu Ferringi x Penang Island, Bukit Bendera)	MALAYSIA (Penang Island, Bayan Lepas)
Exp. 2	sp. 1 x sp. 1	6	6	1	1	1	MALAYSIA (Penang Island, Bayan Lepas)	MALAYSIA (Penang Island, Bayan Lepas)
Exp. 2	sp. 1 x sp. 2	9	42	1	1	0	MALAYSIA (Penang Island, Bukit Bendera x Pahang, Kuantan)	MALAYSIA (Penang Island, Sungai Nipah)
Exp. 2	sp. 1 x sp. 2	6	18	1	1	0	MALAYSIA (Penang Island, Batu Ferringi x Penang Island, Bukit Bendera)	MALAYSIA (Penang Island, Sungai Nipah)
Exp. 2	sp. 1 x sp. 2	3	9	1	1	0	MALAYSIA (Penang Island, Bayan Lepas)	MALAYSIA (Penang Island, Sungai Nipah)
Exp. 2	sp. 1 x sp. 2	7	Wild-caught	1	1	0	MALAYSIA (Penang Island, Bayan Lepas)	MALAYSIA (Penang Island, Sungai Nipah)
Exp. 2	sp. 1 x sp. 2	16	Wild-caught	0	0	0	MALAYSIA (Penang Island, Bayan Lepas)	MALAYSIA (Penang Island, Sungai Nipah)
Exp. 2	sp. 1 x sp. 3	12	9	0	1	0	MALAYSIA (Penang Island, Bukit Bendera x Pahang, Kuantan)	JAPAN (Tokushima, Tokushima)
Exp. 2	sp. 1 x sp. 3	6	3	0	1	0	MALAYSIA (Penang Island, Batu Ferringi x Penang Island, Bukit Bendera)	JAPAN (Tokushima, Tokushima)
Exp. 2	sp. 1 x sp. 3	6	12	1	1	0	MALAYSIA (Penang Island, Bayan Lepas)	JAPAN (Tokushima, Tokushima)
Exp. 2	sp. 1 x sp. 3	6	12	0	1	0	MALAYSIA (Penang Island, Bayan Lepas)	JAPAN (Tokushima, Tokushima)
Exp. 2	sp. 1 x sp. 3	35	Wild-caught	0	0	0	MALAYSIA (Penang Island, Bayan Lepas)	JAPAN (Tokyo, Komae)
Exp. 2	sp. 2 x sp. 1	6	2	0	0	0	MALAYSIA (Penang Island, Sungai Nipah)	MALAYSIA (Penang Island, Bayan Lepas)
Exp. 2	sp. 2 x sp. 1	9	9	1	1	0	MALAYSIA (Penang Island, Sungai Nipah)	MALAYSIA (Penang Island, Bukit Bendera x Pahang, Kuantan)
Exp. 2	sp. 2 x sp. 1	9	18	0	1	0	MALAYSIA (Penang Island, Sungai Nipah)	MALAYSIA (Penang Island, Bayan Lepas)
Exp. 2	sp. 2 x sp. 1	6	21	0	1	0	MALAYSIA (Penang Island, Sungai Nipah)	MALAYSIA (Penang Island, Bayan Lepas)
Exp. 2	sp. 2 x sp. 1	6	Wild-caught	0	1	0	MALAYSIA (Penang Island, Sungai Nipah)	MALAYSIA(Penang Island, Batu Ferringi)
Exp. 2	sp. 2 x sp. 2	8	Wild-caught	1	1	1	MALAYSIA (Penang Island, Sungai Nipah)	MALAYSIA (Penang Island, Sungai Nipah)
Exp. 2	sp. 2 x sp. 2	6	33	1	1	1	MALAYSIA (Penang Island, Sungai Nipah)	MALAYSIA (Penang Island, Sungai Nipah)
Exp. 2	sp. 2 x sp. 2	10	34	1	1	1	MALAYSIA (Penang Island, Sungai Nipah)	MALAYSIA (Penang Island, Sungai Nipah)
Exp. 2	sp. 2 x sp. 2	6	12	1	1	1	MALAYSIA (Penang Island, Sungai Nipah)	MALAYSIA (Penang Island, Sungai Nipah)
Exp. 2	sp. 2 x sp. 2	9	9	1	0	0	MALAYSIA (Penang Island, Sungai Nipah)	MALAYSIA (Penang Island, Sungai Nipah)
Exp. 2	sp. 2 x sp. 3	12	12	1	1	0	MALAYSIA (Penang Island, Sungai Nipah)	JAPAN (Tokushima, Tokushima)
Exp. 2	sp. 2 x sp. 3	30	3	1	1	0	MALAYSIA (Penang Island, Sungai Nipah)	JAPAN (Tokushima, Tokushima)
Exp. 2	sp. 2 x sp. 3	6	27	0	1	0	MALAYSIA (Penang Island, Sungai Nipah)	JAPAN (Tokushima, Tokushima)
Exp. 2	sp. 2 x sp. 3	6	15	1	1	0	MALAYSIA (Penang Island, Sungai Nipah)	JAPAN (Tokushima, Tokushima)
Exp. 2	sp. 2 x sp. 3	20	21	0	1	0	MALAYSIA (Penang Island, Sungai Nipah)	JAPAN (Tokyo, Komae)
Exp. 2	sp. 3 x sp. 1	6	Wild-caught	0	1	0	JAPAN (Tokushima, Tokushima)	MALAYSIA (Penang Island, Bayan Lepas)
Exp. 2	sp. 3 x sp. 1	6	Wild-caught	0	1	0	JAPAN (Tokushima, Tokushima)	MALAYSIA (Penang Island, Bayan Lepas)
Exp. 2	sp. 3 x sp. 1	6	12	0	0	0	JAPAN (Tokushima, Tokushima)	MALAYSIA (Penang Island, Batu Ferringi x Penang Island, Bukit Bendera)
Exp. 2	sp. 3 x sp. 1	18	18	0	0	0	JAPAN (Tokushima, Tokushima)	MALAYSIA (Penang Island, Bayan Lepas)
Exp. 2	sp. 3 x sp. 1	9	13	0	1	0	JAPAN (Tokyo, Komae)	MALAYSIA (Penang Island, Bayan Lepas)
Exp. 2	sp. 3 x sp. 2	6	45	0	0	0	JAPAN (Tokushima, Tokushima)	MALAYSIA (Penang Island, Sungai Nipah)
Exp. 2	sp. 3 x sp. 2	6	24	0	0	0	JAPAN (Tokushima, Tokushima)	MALAYSIA (Penang Island, Sungai Nipah)
Exp. 2	sp. 3 x sp. 2	6	18	0	1	0	JAPAN (Tokushima, Tokushima)	MALAYSIA (Penang Island, Sungai Nipah)
Exp. 2	sp. 3 x sp. 2	6	6	0	0	0	JAPAN (Tokushima, Tokushima)	MALAYSIA (Penang Island, Sungai Nipah)
Exp. 2	sp. 3 x sp. 2	9	27	0	1	0	JAPAN (Tokyo, Komae)	MALAYSIA (Penang Island, Sungai Nipah)
Exp. 2	sp. 3 x sp. 3	6	12	1	1	1	JAPAN (Tokushima, Tokushima)	JAPAN (Tokushima, Tokushima)
Exp. 2	sp. 3 x sp. 3	6	27	1	1	1	JAPAN (Tokushima, Tokushima)	JAPAN (Tokushima, Tokushima)
Exp. 2	sp. 3 x sp. 3	6	6	1	1	1	JAPAN (Tokyo, Komae)	JAPAN (Kanagawa, Yokohama)
Exp. 2	sp. 3 x sp. 3	83	Wild-caught	1	1	1	JAPAN (Tokushima, Tokushima)	JAPAN (Kanagawa, Yokohama)
Exp. 2	sp. 3 x sp. 3	6	27	1	1	1	JAPAN (Kanagawa, Yokohama)	JAPAN (Tokyo, Komae)

*1 = inseminated, 0 = not inseminated.

**1 = eggs laid, 0 = eggs not laid.

***1 = hatchlings produced, 0 = hatchlings not produced.
