# Supplementary material for: ﻿Identification and reproductive isolation of Euborellia species (Insecta, Dermaptera, Anisolabididae) from East and Southeast Asia
Source: Zookeys. 2023 Feb 7;1146:115–34. doi: 10.3897/zookeys.1146.98248 (PMC10194414; doi:10.3897/zookeys.1146.98248)
Supplement: Supplementary material 2 — The specimens and results of morphological measurements [file zookeys-1146-115_article-98248__-s002.pdf]

Table S2. The specimens and results of morphological measurements.

Species	Specimen ID	Sex	Specimen type	Collection site (Origin)	Indication in Figure 3	Measurements (mm) Head width	Pronotum width	Left hindtibia length	Right hindtibia length
Euborellia sp. 1	sp1_19	Female	Wild-caught	MALAYSIA (Penang Island, Batu Ferringi)	Penang-2	1.404	1.326	NA	1.274
Euborellia sp. 1	sp1_20	Female	Wild-caught	MALAYSIA (Penang Island, Batu Ferringi)	Penang-2	1.378	1.300	1.066	NA
Euborellia sp. 1	sp1_21	Male	Wild-caught	MALAYSIA (Penang Island, Bayan Lepas)	Penang-1	1.222	1.222	1.300	NA
Euborellia sp. 1	sp1_22	Female	Wild-caught	MALAYSIA (Penang Island, Bayan Lepas)	Penang-1	1.326	1.222	NA	1.170
Euborellia sp. 1	sp1_23	Female	Wild-caught	MALAYSIA (Penang Island, Batu Ferringi)	Penang-2	1.404	1.248	1.248	NA
Euborellia sp. 1	sp1_24	Male	Wild-caught	MALAYSIA (Penang Island, Bayan Indah beach)	Penang-3	1.378	1.196	1.118	NA
Euborellia sp. 3	sp3_1	Male	Lab. reared offspring	JAPAN (Hyogo, Takasago)	Hyogo	1.326	1.404	1.326	NA
Euborellia sp. 3	sp3_2	Female	Lab. reared offspring	JAPAN (Hyogo, Takasago)	Hyogo	1.560	1.664	NA	NA
Euborellia sp. 3	sp3_3	Male	Lab. reared offspring	JAPAN (Hyogo, Takasago)	Hyogo	1.430	1.508	1.508	1.482
Euborellia sp. 3	sp3_4	Male	Lab. reared offspring	JAPAN (Hyogo, Takasago)	Hyogo	1.378	1.378	1.326	1.326
Euborellia sp. 3	sp3_5	Male	Lab. reared offspring	JAPAN (Hyogo, Takasago)	Hyogo	1.456	1.586	1.378	1.404
Euborellia sp. 3	sp3_6	Male	Lab. reared offspring	JAPAN (Hyogo, Takasago)	Hyogo	1.456	1.560	1.508	1.482
Euborellia sp. 3	sp3_7	Male	Lab. reared offspring	JAPAN (Hyogo, Takasago)	Hyogo	1.274	1.378	1.274	1.274
Euborellia sp. 3	sp3_8	Male	Lab. reared offspring	JAPAN (Hyogo, Takasago)	Hyogo	1.352	1.404	1.326	1.326
Euborellia sp. 3	sp3_9	Female	Lab. reared offspring	JAPAN (Hyogo, Takasago)	Hyogo	1.560	1.664	1.612	1.612
Euborellia sp. 3	sp3_10	Female	Lab. reared offspring	JAPAN (Hyogo, Takasago)	Hyogo	1.664	1.742	1.612	1.638
Euborellia sp. 3	sp3_11	Male	Lab. reared offspring	JAPAN (Hyogo, Takasago)	Hyogo	1.482	1.586	1.534	1.534
Euborellia sp. 3	sp3_12	Male	Lab. reared offspring	JAPAN (Hyogo, Takasago)	Hyogo	1.378	1.456	1.352	1.352
Euborellia sp. 3	sp3_13	Female	Lab. reared offspring	JAPAN (Hyogo, Takasago)	Hyogo	1.534	1.586	1.456	1.508
Euborellia sp. 3	sp3_14	Male	Lab. reared offspring	JAPAN (Hyogo, Takasago)	Hyogo	1.430	1.534	1.482	1.482
Euborellia sp. 3	sp3_15	Male	Wild-caught	JAPAN (Tokushima, Naruto)	Tokushima	1.404	1.508	1.430	1.430
Euborellia sp. 3	sp3_16	Male	Lab. reared offspring	JAPAN (Hyogo, Takasago)	Hyogo	1.508	1.586	NA	1.534
Euborellia sp. 3	sp3_17	Female	Lab. reared offspring	JAPAN (Hyogo, Takasago)	Hyogo	1.768	1.846	NA	1.690
Euborellia sp. 3	sp3_18	Male	Lab. reared offspring	JAPAN (Hyogo, Takasago)	Hyogo	1.560	1.690	1.352	1.560
Euborellia sp. 3	sp3_19	Male	Lab. reared offspring	JAPAN (Kanagawa, Yokohama)	Kanagawa	1.482	1.534	NA	NA
Euborellia sp. 3	sp3_20	Female	Lab. reared offspring	JAPAN (Kanagawa, Yokohama)	Kanagawa	1.664	1.716	NA	NA
Euborellia sp. 3	sp3_21	Female	Lab. reared offspring	JAPAN (Kanagawa, Yokohama)	Kanagawa	1.690	1.846	NA	NA
Euborellia sp. 3	sp3_22	Male	Lab. reared offspring	JAPAN (Kagoshima, Satsuma-sendai)	Kagoshima-1	1.300	1.222	1.274	NA
Euborellia sp. 3	sp3_23	Male	Lab. reared offspring	JAPAN (Kagoshima, Satsuma-sendai)	Kagoshima-1	1.352	1.352	1.352	NA
Euborellia sp. 3	sp3_24	Female	Lab. reared offspring	JAPAN (Kagoshima, Satsuma-sendai)	Kagoshima-1	1.508	1.612	1.430	1.430
Euborellia sp. 3	sp3_25	Female	Lab. reared offspring	JAPAN (Kagoshima, Satsuma-sendai)	Kagoshima-1	1.456	1.508	1.404	NA
Euborellia sp. 3	sp3_26	Male	Lab. reared offspring	JAPAN (Kagoshima, Satsuma-sendai)	Kagoshima-1	1.378	1.352	1.300	NA
Euborellia sp. 3	sp3_27	Male	Lab. reared offspring	JAPAN (Kagoshima, Shimo-koshiki Island)	Kagoshima-2	1.508	1.638	1.534	1.560
Euborellia sp. 3	sp3_28	Male	Lab. reared offspring	JAPAN (Kagoshima, Shimo-koshiki Island)	Kagoshima-2	1.456	1.456	1.430	1.430
Euborellia sp. 3	sp3_29	Male	Lab. reared offspring	JAPAN (Kagoshima, Shimo-koshiki Island)	Kagoshima-2	1.326	1.300	NA	1.274
Euborellia sp. 3	sp3_30	Male	Lab. reared offspring	JAPAN (Shizuoka, Aoi)	Shizuoka-1	1.326	1.274	1.196	1.248
Euborellia sp. 3	sp3_31	Male	Lab. reared offspring	JAPAN (Shizuoka, Aoi)	Shizuoka-1	1.378	1.274	1.222	1.248
Euborellia sp. 3	sp3_32	Male	Lab. reared offspring	JAPAN (Shizuoka, Aoi)	Shizuoka-1	1.378	1.326	NA	1.300
Euborellia sp. 3	sp3_33	Female	Lab. reared offspring	JAPAN (Shizuoka, Aoi)	Shizuoka-1	1.560	1.586	1.560	1.534
Euborellia sp. 3	sp3_34	Female	Lab. reared offspring	JAPAN (Shizuoka, Aoi)	Shizuoka-1	1.560	1.560	NA	1.560
Euborellia sp. 3	sp3_35	Male	Lab. reared offspring	JAPAN (Shizuoka, Izunokuni)	Shizuoka-2	1.482	1.430	1.404	1.404
Euborellia sp. 3	sp3_36	Male	Lab. reared offspring	JAPAN (Shizuoka, Izunokuni)	Shizuoka-2	1.482	1.482	1.378	1.378
Euborellia sp. 3	sp3_37	Male	Lab. reared offspring	JAPAN (Shizuoka, Izunokuni)	Shizuoka-2	1.534	1.612	1.560	NA
Euborellia sp. 3	sp3_38	Male	Lab. reared offspring	JAPAN (Shizuoka, Izunokuni)	Shizuoka-2	1.404	1.430	1.430	1.404
Euborellia sp. 3	sp3_39	Male	Lab. reared offspring	JAPAN (Shizuoka, Izunokuni)	Shizuoka-2	1.378	1.404	1.274	1.274
Euborellia sp. 3	sp3_40	Female	Lab. reared offspring	JAPAN (Shizuoka, Izunokuni)	Shizuoka-2	1.716	1.820	1.638	1.638
Euborellia sp. 3	sp3_41	Male	Lab. reared offspring	JAPAN (Fukushima, Iwaki)	Fukushima	1.378	1.352	1.378	1.378
Euborellia sp. 3	sp3_42	Male	Lab. reared offspring	JAPAN (Fukushima, Iwaki)	Fukushima	1.404	1.404	NA	1.352
Euborellia sp. 3	sp3_43	Male	Lab. reared offspring	JAPAN (Fukushima, Iwaki)	Fukushima	1.352	1.300	1.378	1.352
Euborellia sp. 3	sp3_44	Male	Lab. reared offspring	JAPAN (Fukushima, Iwaki)	Fukushima	1.430	1.404	1.404	1.352
Euborellia sp. 3	sp3_45	Male	Lab. reared offspring	JAPAN (Fukushima, Iwaki)	Fukushima	1.404	1.326	1.378	NA
Euborellia sp. 3	sp3_46	Male	Lab. reared offspring	JAPAN (Fukushima, Iwaki)	Fukushima	1.560	1.768	1.560	NA
Euborellia sp. 3	sp3_47	Female	Lab. reared offspring	JAPAN (Fukushima, Iwaki)	Fukushima	1.612	1.612	NA	1.560
Euborellia sp. 3	sp3_48	Female	Lab. reared offspring	JAPAN (Fukushima, Iwaki)	Fukushima	1.482	1.456	1.404	1.404
Euborellia sp. 3	sp3_49	Female	Lab. reared offspring	JAPAN (Fukushima, Iwaki)	Fukushima	1.768	1.742	1.794	1.820
Euborellia sp. 1 (Ogasawara)	spO_1	Female	Wild-caught	JAPAN (Ioto Island, Ogasawara Islands, Tokyo)	Ioto Island	1.326	1.404	1.274	NA
Euborellia sp. 1 (Ogasawara)	spO_2	Female	Wild-caught	JAPAN (Ioto Island, Ogasawara Islands, Tokyo)	Ioto Island	1.378	1.300	1.092	NA
Euborellia sp. 1 (Ogasawara)	spO_3	Male	Wild-caught	JAPAN (Ioto Island, Ogasawara Islands, Tokyo)	Ioto Island	1.144	1.092	NA	1.118
Euborellia annulata		Female	Wild-caught	French West Indies [Guadeloupe Archipelago] (Basse-Terre Island, Jarry)	Basse-Terre Island	1.430	1.352	1.196	NA
