# Supplementary material for: ﻿Identification and reproductive isolation of Euborellia species (Insecta, Dermaptera, Anisolabididae) from East and Southeast Asia
Source: Zookeys. 2023 Feb 7;1146:115–34. doi: 10.3897/zookeys.1146.98248 (PMC10194414; doi:10.3897/zookeys.1146.98248)
Supplement: Supplementary material 3 — Species and the collection sites of the Dermaptera samples sequenced in this study [file zookeys-1146-115_article-98248__-s003.pdf]

Table S3. Species and the collection sites of the Dermaptera samples sequenced in this study.

DDBJ/ENA/GenBank Accession No.	Specimen_code	Species	Family	Collection site
LC715955	2021BCDNA01	*Euborellia pallipes* (Shiraki, 1905) (= *Euborellia* sp. 3)	Anisolabididae	Japan:Hyogo, Takasago (34.75 N 134.80 E)
LC715956	2021BCDNA02	*Euborellia pallipes* (Shiraki, 1905) (= *Euborellia* sp. 3)	Anisolabididae	Japan:Fukushima, Iwaki (36.88 N 140.79 E)
LC715957	2021BCDNA03	*Euborellia pallipes* (Shiraki, 1905) (= *Euborellia* sp. 3)	Anisolabididae	Japan:Kagoshima, Shimokoshiki Island (31.66 N 129.72 E)
LC715958	2021BCDNA64	*Euborellia pallipes* (Shiraki, 1905) (= *Euborellia* sp. 3)	Anisolabididae	Japan:Shizuoka, Aoi (35.01 N 138.39 E)
LC715959	2021BCDNA10	*Euborellia philippinensis* Srivastava, 1979 (= *Euborellia* sp. 2)	Anisolabididae	Malaysia:Penang Island, Sungai Nipah (5.32 N 100.20 E)
LC715960	2021BCDNA11	*Anisolabis maritima* (Bonelli, 1832)	Anisolabididae	Japan:Tokushima, Naruto (34.20 N 134.60 E)
LC715961	2021BCDNA12	*Anisolabis seirokui* Nishikawa, 2008	Anisolabididae	Japan:Tokushima, Naruto (34.20 N 134.60 E)
LC715962	2021BCDNA15	*Anisolabella ryukyuensis* (Nishikawa, 1969)	Anisolabididae	Japan:Okinawa, Nago (26.57 N 128.02 E)
LC715963	2021BCDNA16	*Gonolabis distincta* (Nishikawa, 1969)	Anisolabididae	Japan:Okinawa, Naha (26.22 N 127.71 E)
LC715964	2021BCDNA19	*Labidura riparia* (Pallas, 1773)	Labiduridae	Malaysia:Penang Island, Bayan Indah beach (5.34 N 100.31 E)
LC715966	2021BCDNA21	*Allostethus indicum* (Burmeister, 1838)	Labiduridae	Malaysia:Penang Island, Bukit Jambul (5.34 N 100.28 E)
LC715967	2021BCDNA25	*Chaetospania javana* Borelli, 1926	Spongiphoridae	Malaysia:Penang Island, Bukit Jambul (5.34 N 100.28 E)
LC715968	2021BCDNA28	*Marava arachidis* (Yersin, 1860)	Spongiphoridae	Malaysia:Penang Island, Minden (5.35 N 100.30 E)
LC715969	2021BCDNA30	*Pseudovostox brindlei* Srivastava, 2003	Spongiphoridae	Malaysia:Penang Island, Bukit Kukus (5.35 N 100.28 E)
LC715971	2021BCDNA35	*Echinosoma sumatranum* (de Haan, 1842)	Pygidicranidae	Malaysia:Penang Island, Bukit Jambul (5.34 N 100.28 E)
LC715972	2021BCDNA40	*Paralabellula curvicauda* (Motschulsky, 1863)	Spongiphoridae	Malaysia:Penang, Bukit Mertajam (5.35 N 100.49 E)
LC715976	2021BCDNA52	*Gonolabis miyatakei* Nishikawa, 2021	Anisolabididae	Japan:Kagoshima, Amami Oshima Island (28.26 N 129.42 E)
LC715977	2021BCDNA58	*Nesogaster amoenus* (Stål, 1855)	Spongiphoridae	Malaysia:Penang Island, Bukit Kukus (5.35 N 100.28 E)
LC715978	2021BCDNA61	*Platylabia major* Dohrn, 1867	Anisolabididae	Malaysia:Penang Island, Bukit Jambul (5.34 N 100.28 E)
LC715979	2021BCDNA62	*Euborellia philippinensis* Srivastava, 1979 (= *Euborellia* sp. 2)	Anisolabididae	Malaysia:Penang Island, Sungai Nipah (5.32 N 100.20 E)
LC715980	2021BCDNA66	*Anisolabella marginalis* (Dohrn, 1864)	Anisolabididae	Japan:Kanagawa, Yokohama (35.52 N 139.64 E)
LC715982	2021BCDNA74	*Gonolabis distincta* (Nishikawa, 1969)	Anisolabididae	Japan:Okinawa, Naha (26.22 N 127.71 E)
LC715985	2021BCDNA77	*Anisolabella marginalis* (Dohrn, 1864)	Anisolabididae	Japan:Tokyo, Komae (35.63 N 139.56 E)
LC715987	2021BCDNA67	*Euborellia annulata* (Fabricius, 1793) (= *Euborellia* sp. 1)	Anisolabididae	Malaysia:Penang Island, Bayan Lepas (5.33 N 100.31 E)
LC715988	2021BCDNA68	*Euborellia annulata* (Fabricius, 1793) (= *Euborellia* sp. 1)	Anisolabididae	Malaysia:Penang Island, Tanjong Bungah (5.47 N 100.24 E)
LC715989	2021BCDNA69	*Euborellia annulata* (Fabricius, 1793) (= *Euborellia* sp. 1)	Anisolabididae	Malaysia:Penang Island, Bukit Bendera (5.42 N 100.26 E)
LC715990	2021BCDNA70	*Euborellia annulata* (Fabricius, 1793) (= *Euborellia* sp. 1)	Anisolabididae	Malaysia:Pahang, Kuantan (3.80 N 103.34 E)
LC715991	2021BCDNA72	*Gonolabis michikoae* Nishikawa, 2021	Anisolabididae	Japan:Kagoshima, Amami Oshima Island (28.31 N 129.31 E)
LC731318	DER19	*Euborellia annulipes* (Lucas, 1847)	Anisolabididae	Japan:Kagoshima, Amami Oshima Island (28.31 N 129.31 E)
LC740580	2021BCDNA91	*Euborellia annulata* (Fabricius, 1793)	Anisolabididae	French West Indies:Jarry, Basse-Terre Island, Guadeloupe Archipelago (16.23 N 61.55 W)
